# Effects of Pilates Training on Physiological and Psychological Health Parameters in Healthy Older Adults and in Older Adults With Clinical Conditions Over 55 Years: A Meta-Analytical Review

**DOI:** 10.3389/fneur.2021.724218

**Published:** 2021-10-25

**Authors:** Lilly Meikis, Pamela Wicker, Lars Donath

**Affiliations:** ^1^Institute of Movement and Sport Gerontology, German Sport University Cologne, Cologne, Germany; ^2^Department of Sports Science, Bielefeld University, Bielefeld, Germany; ^3^Institute of Exercise Science and Sport Informatics, German Sport University Cologne, Cologne, Germany

**Keywords:** Pilates, rehabilitation, exercise therapy, mind-body intervention, fitness

## Abstract

This meta-analytical review aimed at comparing the impact of Pilates interventions (PIs) on physiological and psychological health parameters in healthy older adults and older adults with a clinical condition aged 55 years and older. The literature search was conducted in three databases (PubMed, Web of Science, SPORTDiscus). Randomized controlled trials that aimed at improving physiological and psychological health parameters in adults aged 55 years and older using Pilates as an intervention were screened for eligibility. The included data was extracted and assigned based on participants' health condition (clinical vs. non-clinical), as well as the respective control condition used in the study [inactive (IC) vs. active control group (AC)]. Statistical analyses were computed using a random-effects inverse-variance model. Fifty-one studies with a total of 2,485 participants (mean age: 66.5 ± 4.9 years) were included. Moderate effects (SMD: 0.55; 0.68) were found for physiological health parameters (muscle strength, balance, endurance, flexibility, gait, and physical functioning) in both experimental (clinical and non-clinical) conditions when compared to ICs (*p* < 0.003; *p* = 0.0001), and small to moderate effects (SMD: 0.27; 0.50) when compared to ACs (*p* = 0.04; *p* = 0.01). Moderate to large effects (SMD: 0.62; 0.83) were documented for psychological health parameters (quality of life, depression, sleep quality, fear of falling, pain, and health perception) in both conditions when compared to ICs (*p* < 0.001, *p* < 0.001). PIs induce small to large effects in physiological and psychological health parameters in older adults, regardless of their health condition. The substantial heterogeneity within the included studies complicated standardized comparison of the training modalities between the two target groups. Nonetheless, Pilates seems to be a safe, adaptable, and promising exercise approach for a heterogenous population of older adults.

## Introduction

The aging population has grown over the last century (1). The public health care systems are burdened of physical and psychological dysfunctioning of older populations due to age-related declines and illnesses (2). In addition, the older population will double in the next few years. The so-called baby boomer generation (born from 1946 to 1964) is the main contributor to this growth (3). Accordingly, social, economic, and health problems are becoming critical (4). Main characteristics for the biological and heterogeneous aging process are declines in physical and cognitive functions, such as a reduced muscle mass and strength, a lower aerobic capacity, a loss in brain volume, and function and a decrease in bone density (5, 6). The decline of physical and cognitive functions often implies additional psychological changes due to fear, disabilities, lower self-confidence, or loss of autonomy (7). Thus, the physiological and psychological health status of older adults are highly interdependent (8, 9). The health promotion of these older individuals (>55 years) is, therefore, essential and aging should not be dominated by frailty or disability. Healthy aging is characterized by the maintenance of independence as well as the prevention of diseases (10). Participation in physical activity has the effect of preventing or alleviating diseases such as cardiovascular diseases, Type II Diabetes, obesity, cancer, depression, and Alzheimer's disease (11) and further improves psychological health (12–14). Therefore, it is essential to promote physical activity, and to maintain the independence and quality of life of older adults (15). In this context, exercise-based strategies are fundamental for increasing or maintaining general activity levels (5, 16, 17). Holistic training approaches are designed to develop overall health. While their goal is not to improve isolated health aspects, they have the purpose of enhancing individuals' general condition (18). Within holistic strategies, Pilates recently emerged as a popular method and continues to gain interest (19, 20). This method is used in the field of rehabilitation and fitness to facilitate improvements in strength, flexibility, balance, endurance, coordination, and psychological health status (2, 21, 22).

Pilates was originally developed by Joseph H. Pilates and follows the eight principles of flow of movements, centering, control, breathing, range of motion, precision, stability, and opposition (20, 23). In the early 1920s, the physical benefits of the Pilates method led Josef H. Pilates to train dancers who were prone to injury. His practice did not only improve their performance, but also shortened the recovery time of injured dancers (24). Several positive effects as well as the easy adaptation of Pilates exercises to different target groups make Pilates an appealing and effective rehabilitation and prevention approach (25). In terms of exercise, Pilates can be performed on a mat or with specific equipment (e.g.,: Ladder Barrel, Reformer, Pilates Trapeze) (26). The level of difficulty and load may be individually varied by participants, for example by adjusting springs, changing positions, or using small equipment (e.g., rubber bands, Swiss Balls). The exercises primarily address the deep muscle strength and flexibility, while building awareness between body, movement, and mind – according to the Pilates principles (23, 27). Pilates can, hence, be classified as a mind-body intervention (26). According to the literature, there is no age limit for Pilates. In fact, it was found to be particularly suitable and safe for older adults (28). Pilates is increasing in popularity as a valuable intervention for older adults due to its well-established positive impact on physiological and psychological health parameters, especially in this age group. Various meta-analyses reported benefits with regard to flexibility, muscle tone and strength, balance, coordination, postural control, aerobic endurance, body composition, and functional autonomy (1, 2, 20). The systematic review of Bullo et al. (19), consisting of ten randomized-controlled trials (RCTs), documented relevant effects on the improvement of lower-limb strength, dynamic balance, and walking/gait assessment in older adults after Pilates intervention (PI). Small and positive effects were detected regarding static balance and flexibility. Also, Pilates was shown to help maintain independence and improve mood state and quality of life (19). Additionally, Barker et al. (29) stated that PIs led to an increment of dynamic and static balance abilities.

While the effects on balance ability have been widely studied (19, 23, 29–31), other parameters have been examined to a lesser extent (e.g., aerobic endurance) (20). In the current literature, reviews mainly focused on the measurement of physiological health parameters. However, reviews and research on Pilates and its influence on psychological factors are scarce, especially in the context of older adults (32).

Due to the well-documented efficacy of Pilates, it is not only used as a fitness approach, but also as a rehabilitation tool (25). In the clinical context, the effectiveness of PIs was investigated in middle-aged women dealing with breast cancer (33), the most common kind of cancer in older women (34, 35). Five RCTs and two non-RCTs were included in a meta-analysis (33). Positive effects of PIs particularly affected a number of psychological parameters, including quality of life, pain, mood, and self-reported upper extremity function. Larger effects compared to traditional training groups were observed with respect to improving pain symptoms and self-reported upper extremity function (33). Besides cancer, aging is a major influencing factor for several non-communicable diseases (NCD) like chronic obstructive pulmonary disease, cardiovascular disease, Type II Diabetes, cognitive decline, and Dementia (36). It is estimated that more than 50% of older adults must deal with two or more chronic diseases and their adverse consequences (36, 37). Hence, there is a need for interventions that focus on more than one chronic disease (37) and adapt to individuals' age and health status (38). In the adult population, it is evident that PIs result in higher exercise tolerance, muscle strength, and health-related quality of life in patients with NCD. Still, further research is necessary to clarify clinical effectiveness and establish the most suitable intervention protocol for PIs (39). In addition to NCD, PIs led to significant and positive effects in patients with Parkinson's disease. Parkinson's is a common disease that occurs particularly in older adults and causes motor dysfunctions (40). Specifically, it causes gait impairments and postural control ability and leads to a higher risk of falling or other health problems, ultimately aggravating age-related declines (41). Thus, movement therapies are crucial methods, since drug therapies or surgical interventions cannot eliminate all impairments (40). Pilates was found to be a safe exercise strategy for patients with Parkinson's: In a recent meta-analysis (42), PIs led to beneficial effects in overall fitness, balance, and functional autonomy. Compared to other exercise approaches, Pilates was more effective in improving lower-body functions (42).

Collectively, Pilates was found to be a suitable and effective movement-based rehabilitation approach (33, 39, 42), especially due to its individual adaptability and the safeness of its exercises (27, 28). However, further research is necessary for a better understanding of the clinical effectiveness of Pilates (43, 44) and, additionally, for a better state of evidence on mental health outcomes (44). There is a lack of reviews examining the overall (clinical) effectiveness of PIs on physiological and psychological health parameters in older adults. Based on the different applications of PIs and the outlined gaps in the literature, the intentions of this meta-analysis were:

- To calculate and classify the effects of Pilates interventions on physiological and psychological health parameters in healthy older adults and older adults with clinical conditions aged 55 years and older;
- To compare the intervention effects with inactive and active control groups;
- To compare the intervention effects and training characteristics between healthy participants and participants with clinical conditions;
- To provide further research recommendations in the field of Pilates intervention with adults aged 55 years and older.

## Methods

### Search Strategy and Study Selection

This meta-analytical review was performed according to the PRISMA guidelines (45). The literature search was conducted by two independent researchers in three sports, health-related, and biomedical databases (SPORTDiscus, Web of Knowledge, and PubMed RRID: SCR_000512). The search started on June 19, 2020 and ended on June 25, 2020. A manual research update was conducted between August 24, 2021 and September 4, 2021 for a higher actuality of articles and followed the same process as the first search. Boolean conjunctions (OR/AND) were applied to combine the relevant search terms (operators). The search was structured according to three search levels (Table 1). In addition, citation tracking and a manual search were carried out to identify important primary articles, which however did not produce any additional results. The studies underwent a manual screening process after duplicates were removed. The screening process was divided into three phases: (1) title, (2) abstract, and (3) full-text and followed several in- and exclusion criteria. The inclusion criteria were established according to the PICOS scheme: population (P), intervention (I), comparators (C), outcomes (O), and study design (S) (45). If the study had no title at all (e.g., symbols, numbers, etc.), a remaining duplicate was detected, or the study was stated as being a review, it was removed. If a deviation between both examiners occurred, a further re-screening was conducted and both reviewers reached consensus after a discussion. All relevant articles were in German or English.

**Table 1:** Search levels and search terms of the literature search process.

**Search level**	**Search terms with Boolean operators**
Search #1	((All Fields) (power OR activity OR strength OR endurance OR coordination OR balance OR mobility OR recovery OR recreation OR health OR “physical fitness” OR “physical function” OR capacity OR function OR psychological OR well-being OR wellness OR “quality of life” OR self-esteem OR self-efficacy OR “fear of falling” OR confidence OR depression OR “depressive symptoms” OR “sleep quality” OR emotional OR “mental health”))
Search #2	#1 AND ((Title) Pilates (Abstract) AND Pilates)
Search #3	#2 AND ((All Fields) (old OR older OR senior OR aged OR “senior citizen” OR “senior citizens” OR seniors OR “old age” OR “older adults”) NOT (child OR children OR “young adults” OR middle-aged OR students OR “college students” OR adolescent))

The following inclusion criteria were applied:

- The full-text article was published in English or German (or a translation was available) in a journal with peer review.
- The mean age of the study sample was >55 (P).
- The Pilates interventions involved equipment- or mat-based exercises and incorporated the principles of Pilates training (I).
- At least one control group, which either received an exercise-based intervention (active control = AC) or did not receive an exercise-based intervention (inactive control = IC) was adopted as a comparator (C).
- Parameters capturing physiological and psychological health (O) were extracted.
- The paper presented a randomized and controlled intervention study with a pre- and post-testing comparison (S) were considered.

The following exclusion criteria were used:

- Pilates intervention was combined with other training approaches, which do not contribute to usual care or supplemental medication intake.
- Pilates intervention lasted <4 weeks or/and less than two training sessions per week were performed.
- No control group was used.
- Measurements of biomechanical, chemical, and microbiological changes, or the measurements of parameters according to cognitive functions and/or body composition were excluded.

### Assessment of Methodological Quality

To assess the methodological quality of the randomized trials, the PEDro (Physiotherapy Evidence Database) scale (46) was used. The PEDro scale, consisting of 11 dichotomous items (yes or no), assessed the internal validity and statistical information of the included studies. The rating process was conducted by two independent researchers. If consensus was not reached on every item, a re-evaluation was performed by the non-blinded researchers together.

### Data Extraction

The relevant data was examined independently by two scholars. The given study information were extracted and then converted to a table format regarding references (authors and year of publication), study design, condition of the sample (clinical vs. non-clinical), sample (age), groups (numbers of participants), characteristics of the PIs' and control groups' interventions (exercises, intensity, and used equipment), training load (frequency, duration, and adherence), outcomes (test names, mean and standard deviations of the pre- and post-test measurements) and PEDro scores (see Supplementary Tables 1, 2). Available data of the pre- and post-test measurements was divided into physiological and psychological health parameters and according to the respective condition of the participants (clinical vs. non-clinical). Additionally, a subdivision was performed to group studies based on whether an active (AC) or inactive control (IC) group available. The change in means from (pre-intervention) baseline to post-intervention was adopted as the physiological and psychological outcome measures. Based on the given study's design, PI groups were either compared to ICs or ACs. The effect sizes and standard error values were pooled together if a study used more than one tool to measure the same outcome variable. Corresponding authors were contacted *via* email to request required missing values of the outcome variable. If the authors still did not respond after a second reminder, the records were excluded. This review focused on PI.

### Statistical Analysis

For each included study, the standardized mean differences [SMD, with 95% confidence intervals (CI)], from pre- to post-tests, were computed separately according to adjusted Hedges' g (47). The difference of the respective outcome between the intervention (PI) and the control condition (IC and AC) including the pooled standard deviations were computed for each relevant physiological and psychological outcome variable separately. These results were divided according to the respective health condition (non-clinical and clinical). Negative effects were signed with a minus sign. If results were presented as figures, the WebPlotDigitizer Version 4 (Free Software Foundation, Boston, MA, RRID: SCR_013996) was used to extract means and standard deviations. The Cochrane Review Manager Software (RevMan 5.3, Cochrane Collaboration, Oxford, UK, SCR_003581) was used to perform statistical analyses and to compute the effect sizes using an inverse-variance approach (48) with a random-effects model (49). The following scale was used to classify the dimension of SMD: 0–0.19 = negligible effect, 0.20–0.49 = small effect, 0.50–0.79 = moderate effect and 0.80 = large effect (50). Potential publication bias was examined with a funnel plot. The significance level α was set at 0.05. Forest plots (95% CI) were generated for each type of health measure (physiological or psychological), control group adopted in the study (inactive or active), and the respective health condition of the participants (clinical or non-clinical).

## Results

### Trial Flow

**Figure 1:**
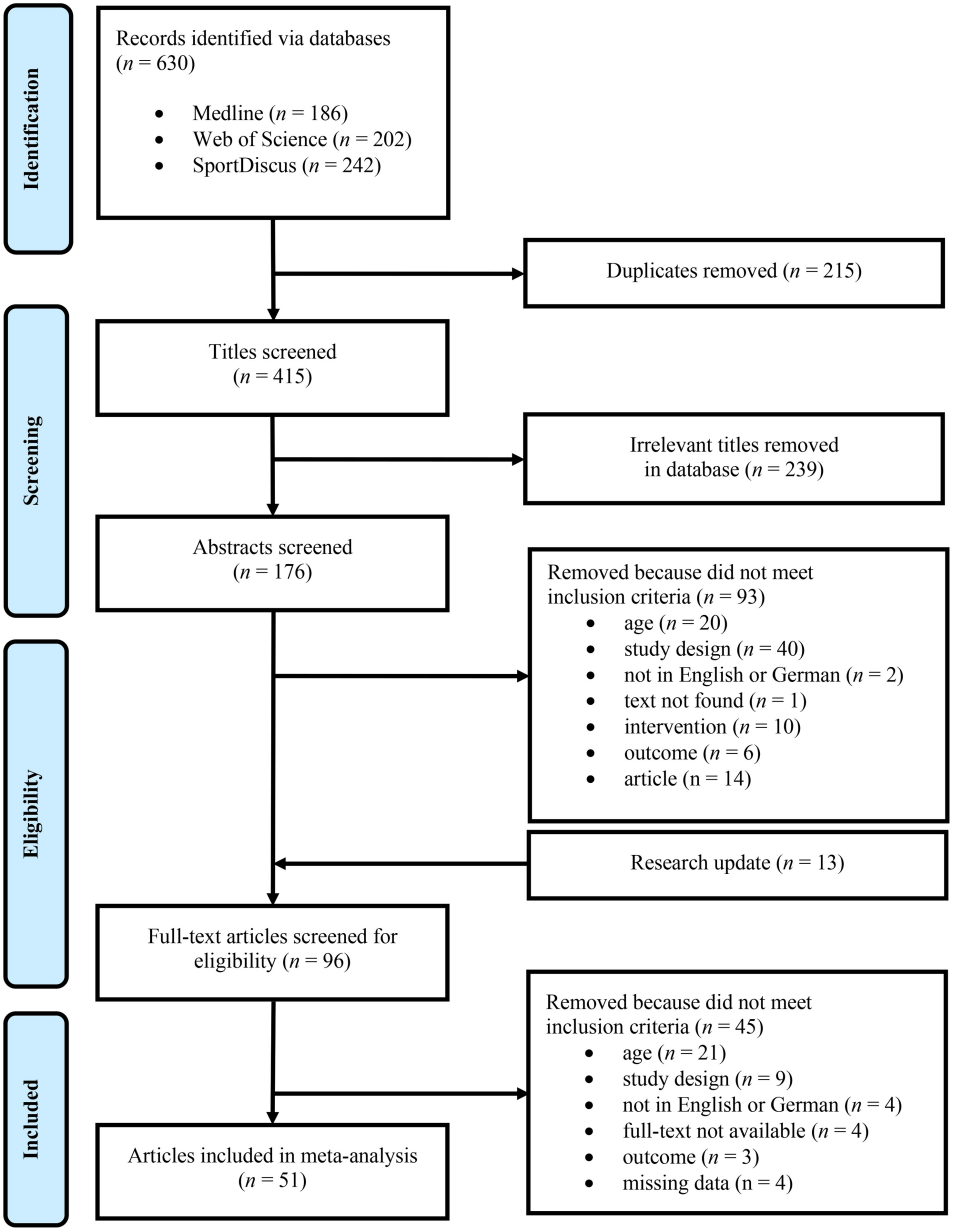
Flowchart demonstrating study screening and selection.

During the search process, 630 potential articles were identified (Figure 1). After removing duplicates, 415 titles were screened for eligibility. After this screening, 176 abstracts remained and were carefully studied regarding the inclusion criteria. After a research update for actuality, another 13 potential studies were included. The remaining 96 full texts were subjected to further review of which 45 were excluded due to exclusion criteria or miss-matching inclusion criteria. Four studies had to be removed due to missing data, meaning a lack of pre- and post-values. Finally, 51 articles were included in the meta-analysis.

### Study Population and Study Quality

#### Participants

Across the 51 included studies, a total of 2,485 participants with a mean age of 66.5 ± 4.9 were considered (51–101). Altogether, 1,105 participants followed a PI, 725 participants were part of an IC group, and 636 joined an AC group. The sample sizes ranged between 19 (76) and 115 (84) with a mean sample size of 49.9 ± 25.7 per study.

Thirty-one trials only included women, whereas one study only included men (69). One third of the studies included participants with a clinical condition (*n* = 18), which were declared as follows: risk of falling (55), women with chronic low back pain (61, 62, 97), participants with Chikungunya Fever (65), men with post-prostatectomy urinary incontinence (69), participants with impaired balance (73), total knee arthroplasty (74), women with Osteoporosis (75, 85), post-stroke patients (76, 77, 90), women with Type II Diabetes (81), individuals with Parkinson's Stage 1–3 (83), knee osteoarthritis in women (99), and women with breast cancer (84, 100).

#### Interventions

Out of 51 studies, 36 studies were two-arm RCTs and 15 three-arm RCTs. All study arms were incorporated in the results of the meta-analysis except for two (53, 84). Yoga intervention served as AC (1) and not the water exercise intervention AC (2). Yoga is also classified as mind-body intervention (102), which is why the comparison to this study arm was preferred. In the other study, inspiratory muscle training was labeled as active control group (53), which seems not conclusive and was therefore excluded as study arm.

Every trial included a PI, either as mat- or equipment-based Pilates. A total of 11 studies performed an equipment-based intervention (53, 55, 57, 58, 73, 78, 86–89, 91) with the following devices: Reformer, Cadillac, Wall Unit, Combo Chair, and Ladder Barrel. In addition to the mat-based Pilates training, small devices were used such as resistance bands, Magic Circles, Fitballs, Gym Sticks, Chi Balls, foam roller, and free weights.

Total and official Pilates sessions varied between 10 (57, 69) and 144 (84), with 24 sessions being the most frequently adopted approach (*n* = 21). The period of interventions ranged from 4 weeks (82, 94) to 1 year (75, 84) of length. The typical study length was 8 or 12 weeks (29 out of 51 studies). Altogether, 27 studies implemented two training sessions per week and 23 studies included three sessions per week. In one case, only one training session was performed per week, but additional home sessions were held daily (69). The training sessions' duration varied between 30 and 66 min, with more than half of the studies using 60 min as the duration of one training session *(n* = 39).

ICs either instructed participants to stay with their normal activity level (51, 52, 56, 57, 59, 60, 66, 67, 71, 78, 82, 85, 87, 89, 93, 96, 101), to not exercise or train (53, 68, 69, 72, 76, 79, 90–92, 94, 95, 99), to take part at a monthly meeting for communication (63, 64), to attend educational workshops (97, 100), or to take part in standard clinical care (65). The ACs' intervention approaches included an unspecific activity program (54, 97), home exercises (for balance and strength) (55, 75), a static stretching program (58, 86, 88), a muscular strength training (59), a resistance training (60), physiotherapy (61, 62), a Proprioceptive Neuromuscular Facilitation Training (82, 94), a traditional balance training (66), pelvic floor muscle training with anal electrical stimulation (69), unstable support surface exercises (70), walking (72), traditional exercising (73), suspension training with TRX (99), general physical activity program (98), clinical rehabilitation (74, 77), Huber Training (80), aerobic exercises (83), Yoga (84, 93), 5 min body vibration (89), Latin Dance sessions (92), and Aqua-Fitness (95). More detailed information is presented in Supplementary Table 1.

#### Interventions With Non-clinical vs. Clinical Conditions

Altogether, 18 out of 51 studies examined participants with clinical conditions (CC). The mean length of interventions including participants with non-clinical conditions (non-CC) was 12.27 ± 5.08 weeks, compared to 13.67 ± 12.80 weeks for interventions including participants with CC. Both intervention types mainly held two 60-min sessions per week, whereas 14 non-CC and 8 CC trials implemented three training sessions per week. One study did not report the duration of the Pilates sessions (74). Nearly half of the non-CC trials (16 out of 33) and five out of 18 CC trials focused exclusively on the comparison to an IC.

In one case, additional home exercises were carried out once a week for the non-CC trials (57). For the CC trials, three studies reported additional home exercises with a frequency of either every day (55, 69), or five times per week (73). Four trials included an additional treatment of either physiotherapy (61, 62), standard clinical care (66), or a rehabilitation program (74).

If the intensity was mentioned within the non-CC trials, it was described as low and moderate (72), moderate to moderate-to-vigorous (59, 60, 88), or a 5–6 on a modified Borg Scale (89). A frequently used training load was one set and ten repetitions per exercise. Exceptions were 2–4 sets (53, 56, 80) and more than 15 repetitions per exercise (80, 98).

Besides individual exercise adaptations, three CC trials adopted training adjustments for clinical suitability (77, 84, 100). The intensity of CC studies was described as light-to-moderate (65), moderate (81), 7 on a modified Borg Scale (83), 12–14 on the Borg Perceived Exertion Scale (73), and 45–60% of the heart rate reserve (84). Repetitions were usually kept under 15 (65, 69, 73, 77, 81, 83) and the number of sets varied between one and three (77, 81, 83).

#### Outcomes and Instruments

The relevant results were categorized into physiological and psychological health parameters. The filtered, physiological health parameters were classified according to muscular strength, balance (static, dynamic), flexibility, endurance, gait quality, and physical functioning measures. Psychological health parameters consisted of measurements of quality of life, depression, sleep quality, fear of falling, pain, fatigue, and health perception. Most of the trials included only physiological measures (32 out of 51). Five studies included only psychological parameters (52, 62, 63, 78, 84), while 16 encompassed both physiological and psychological measures (51, 52, 58, 61, 64, 65, 69, 72–75, 85, 89, 91, 93, 95). Due to the large sample size of the included studies and the consideration of physiological as well as psychological health parameters, the measuring instruments demonstrate substantial heterogeneity (see Supplementary Table 1).

### Risk of Bias Assessment

**Figure 2:**
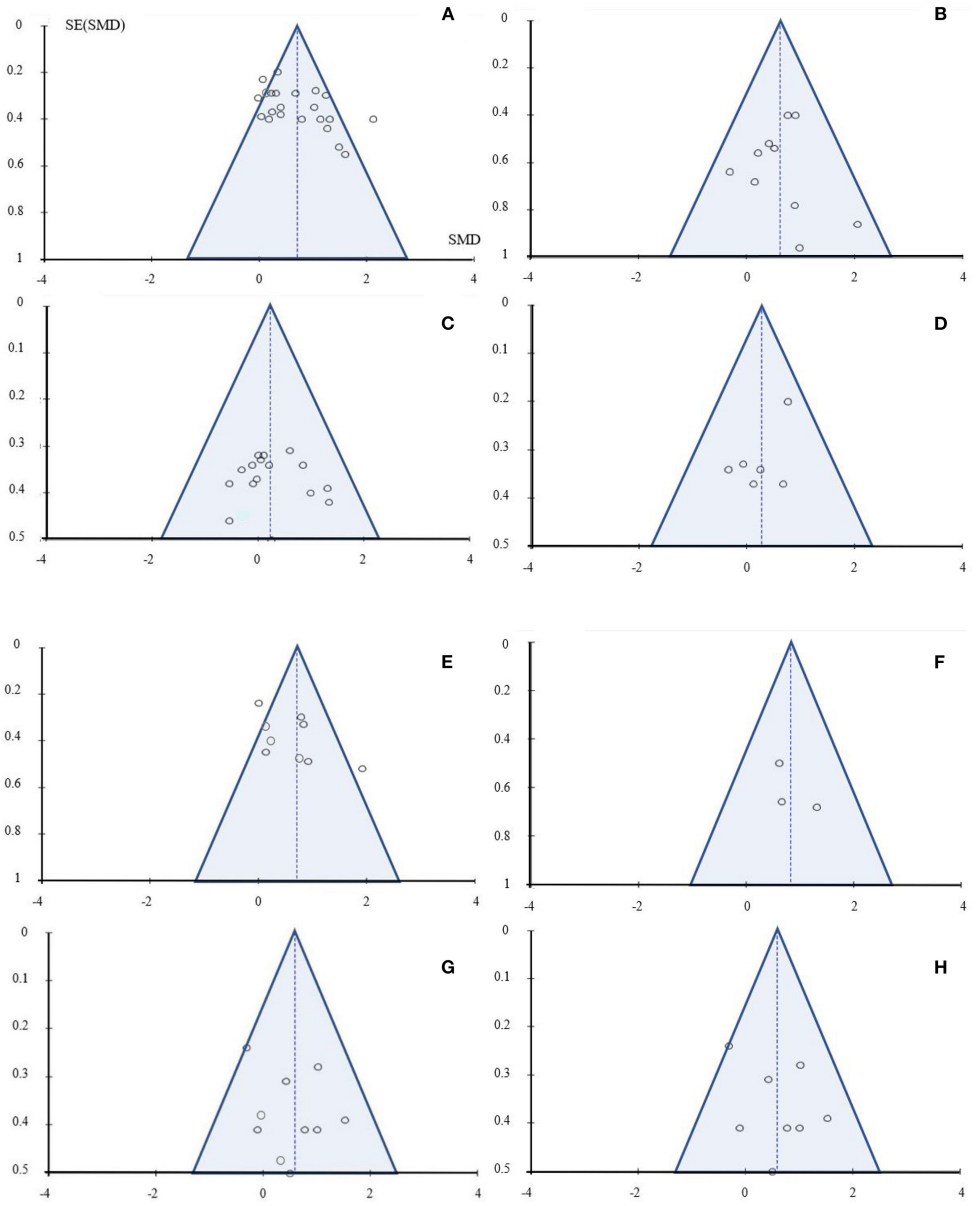
Funnel plot: **(A)** non-clinical condition - physiological health parameters: Pilates vs. IC; **(B)** non-clinical condition - psychological health parameters: Pilates vs. IC; **(C)** non-clinical condition - physiological health parameters: Pilates vs. AC; **(D)** non-clinical condition - psychological health parameters: Pilates vs. AC; **(E)** clinical condition - physiological health parameters: Pilates vs. IC; **(F)** clinical condition - psychological health parameters: Pilates vs. IC; **(G)** clinical condition - physiological health parameters: Pilates vs. AC; **(H)** clinical condition - psychological health parameters: Pilates vs. AC.

**Figure 3:**
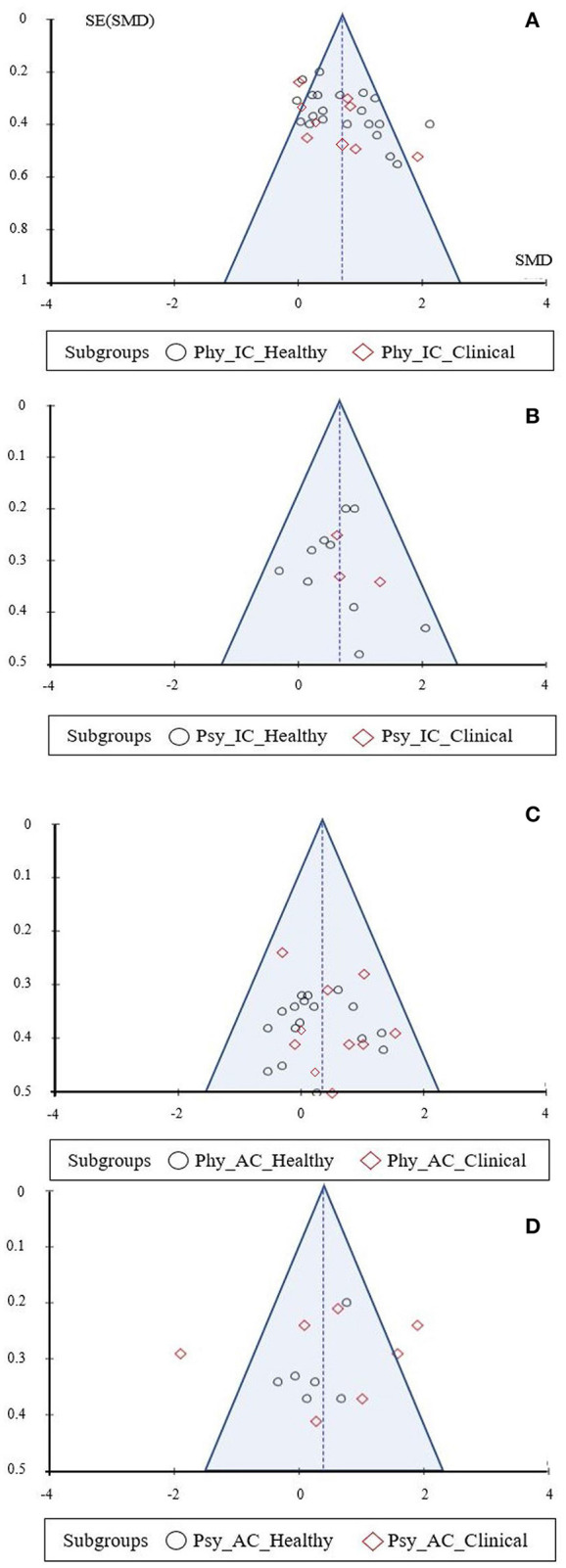
Funnel plot: **(A)** clinical and non-clinical conditions - physiological health parameters: Pilates vs. IC; **(B)** clinical and non-clinical conditions - psychological health parameters: Pilates vs. IC; **(C)** clinical and non-clinical conditions - physiological health parameters: Pilates vs. AC; **(D)** clinical and non-clinical conditions - psychological health parameters: Pilates vs. AC.

Most of the funnel plots did not show a clear funnel-shape (Figures 2, 3), meaning that studies with a higher and/or smaller sample size (different standard error sizes) were missing. Concerning the subgroup analysis (clinical vs. non-clinical condition) and the comparison of Pilates vs. ICs in the physiological parameters, a clear funnel-shape is depicted with the absence of smaller sample size studies (Figure 3A). Also, an imbalance is evident and fewer intervention studies under clinical conditions were included. A similar pattern emerged from the subgroup analysis, Pilates vs. ACs, regarding physiological health parameters, with the absence of high sample size studies (Figure 3C).

According to the PEDro scores, the study quality ranged between 3 (90) and 9 (68, 69, 75, 85, 86) (median of 7). A score of five or lower was labeled as *weaker* and studies with a PEDro score of six and higher were labeled as *stronger*. Six out of 46 studies showed a PEDro score of five or lower (56, 67, 70, 90, 92, 95). Only one study blinded the subjects (66), while the rest of the included trials did not blind the therapists or subjects, as conducting blinded analyses is generally complicated in the context of exercise interventions (Supplementary Table 2).

### Data Analyses of Healthy Participants

#### Pilates vs. Inactive Control

Comparing PIs to ICs in healthy older adults, moderate and significant effects were demonstrated for physiological (muscle strength, balance, endurance, flexibility, and physical functioning) [*p* < 0.001, SMD: 0.68 (95% CI: 0.44, 0.91), l^2^ = 64%] as well as psychological health parameters (quality of life, depression, sleep quality, fear of falling, and health perception) [*p* = 0.0002, SMD: 0.62 (95% CI: 0.30, 0.94), l^2^ = 68%].

#### Pilates vs. Active Control

Comparing PIs to ACs in healthy older adults, small and significant effects were demonstrated for physiological (muscle strength, balance, endurance, flexibility, and physical functioning) [*p* = 0.04, SMD: 0.27 (95% CI: 0.02, 0.53), l^2^ = 55%] and small, non-significant effects for psychological (quality of life, depression, sleep quality, and fear of falling) [*p* = 0.15, SMD: 0.28 (95% CI: −0.10, 0.65), l^2^ = 55%] health outcomes.

### Data Analyses of Participants With Clinical Conditions

#### Pilates vs. Inactive Control

Comparing PIs to ICs in older adults with clinical conditions, moderate and significant effects for physiological health parameters (muscle strength, balance, gait, flexibility, and physical functioning) were found [*p* = 0.003, SMD: 0.55 (95% CI: 0.19, 0.91), l^2^ = 54%]. Regarding psychological health measures (quality of life, pain, and health perception), large and significant effects were evident [*p* < 0.001, SMD: 0.83 (95% CI: 0.42, 1.24), l^2^ = 32%].

#### Pilates vs. Active Control

The comparison of PIs to ACs in the context of participants with a clinical condition showed moderate and significant effects for the physiological health parameters (muscle strength, balance, flexibility, and physical functioning) [*p* = 0.01, SMD: 0.50 (95% CI: 0.10, 0.90), l^2^ = 68%]. Moderate and non-significant effects were identified for the psychological health measures (quality of life, pain, fear of falling) [*p* = 0.28, SMD: 0.51 (95% CI: −0.41, 1.43), l^2^ = 95%].

### Data Analyses Healthy vs. Clinical Conditions

#### Pilates vs. Inactive Control

**Figure 4:**
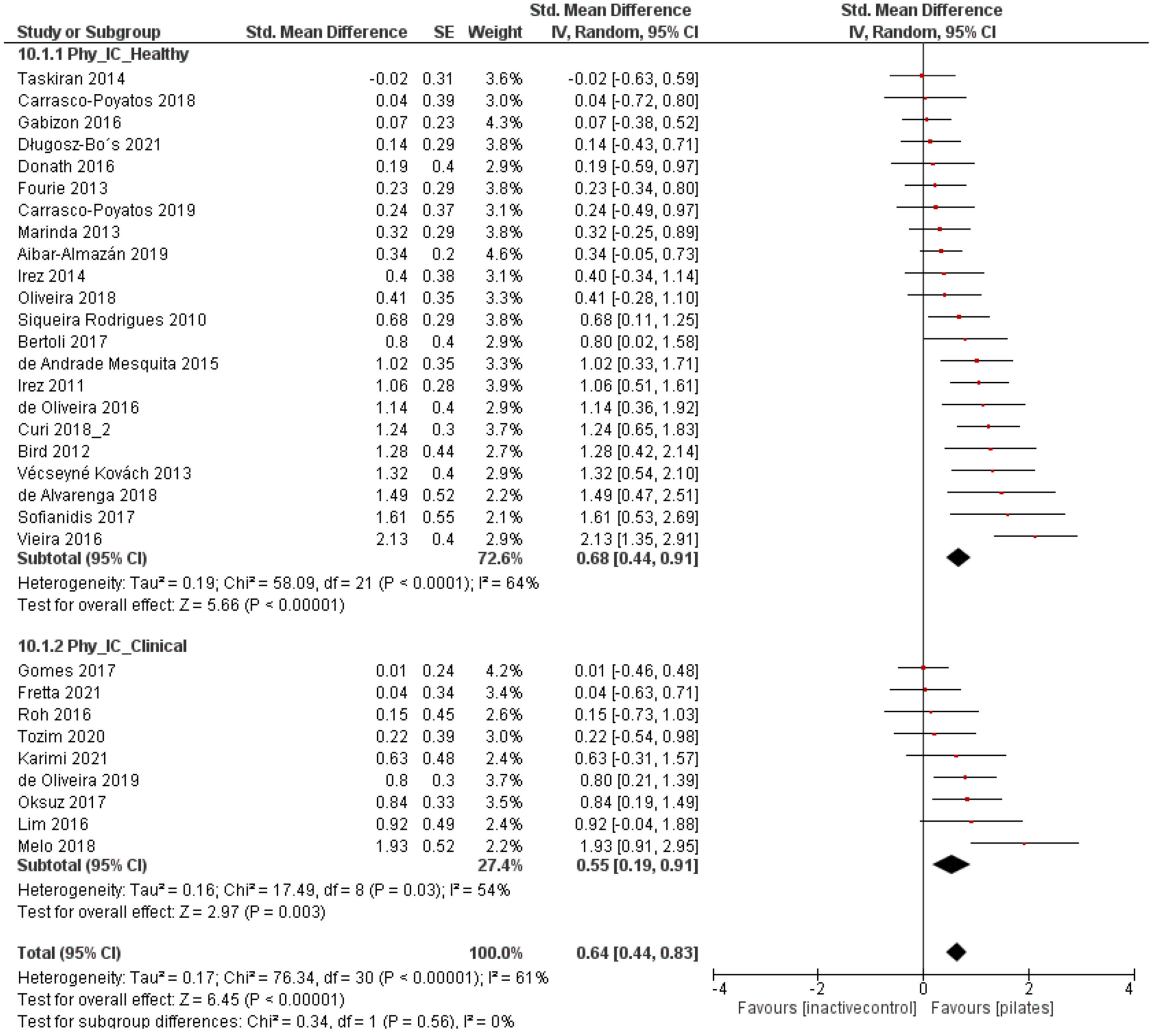
Forest plot of standardized mean effects of Pilates intervention on physiological health parameters compared to inactive control groups. Data are presented for healthy older adults and older adults with clinical conditions. SE, standard error; IV, inverse variance model; CI, confidence interval; Std., standardized.

**Figure 5:**
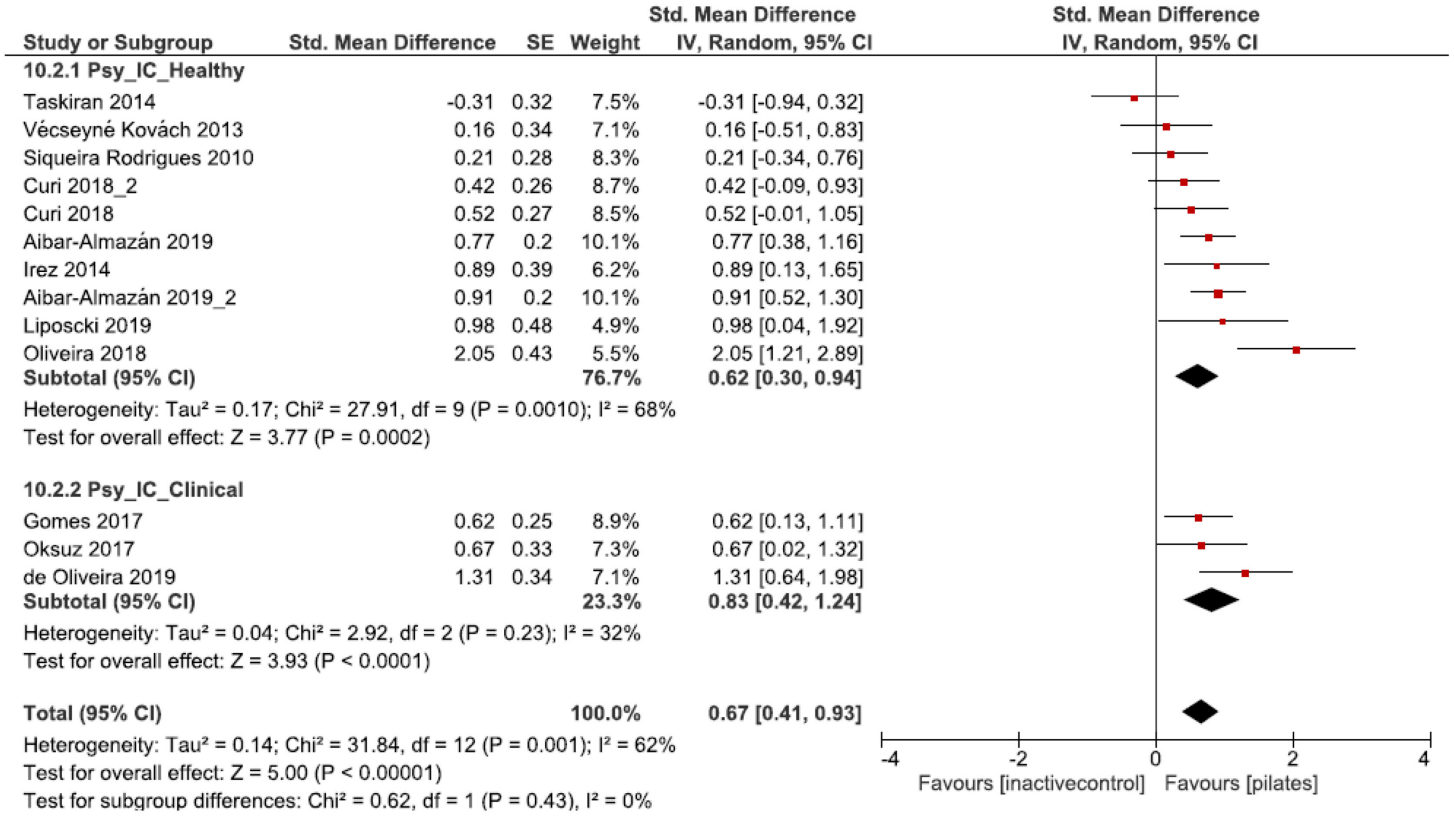
Forest plot of standardized mean effects of Pilates intervention on psychological health parameters compared to inactive control (IC) groups. Data are presented for healthy older adults and older adults with clinical conditions. SE, standard error; IV, inverse variance model; CI, confidence interval; Std., standardized.

Both groups, healthy participants as well as participants with a clinical condition, demonstrated moderate and significant effects [*p* < 0.001, SMD: 0.68 (95% CI: 0.44, 0.91), l^2^ = 64%; *p* = 0.003, SMD: 0.55 (95% CI: 0.19, 0.91), l^2^ = 54% Figure 4] for the physiological health parameters (muscle strength, balance, gait, endurance, flexibility, and physical functioning). No subgroup differences were observed [*p* = 0.56, SMD: 0.64 (95% CI: 0.44, 0.83), l^2^ = 61%; Figure 4]. Considering psychological health parameters (quality of life, depression, sleep quality, fear of falling, health perception, and pain), the subgroups presented moderate to large and significant effect sizes [*p* < 0.001, SMD: 0.62 (95% CI: 0.30, 0.94), l^2^ = 68%; *p* < 0.001, SMD: 0.83 (95% CI: 0.42, 1.24) l^2^ = 32%; Figure 5]. Again, no significant subgroup differences emerged [*p* = 0.43, SMD: 0.70 (95% CI: 0.41, 0.93), l^2^ = 62%; Figure 5].

#### Pilates vs. Active Control

**Figure 6:**
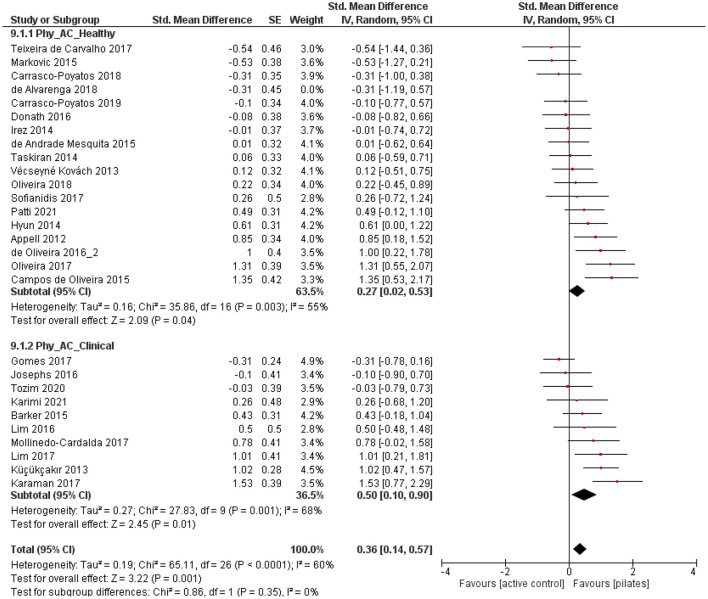
Forest plot of standardized mean effects of Pilates intervention on physiological health parameters compared to active control (AC) groups. Data are presented for healthy older adults and older adults with clinical conditions. SE, standard error; IV, inverse variance model; CI, confidence interval; Std., standardized.

**Figure 7:**
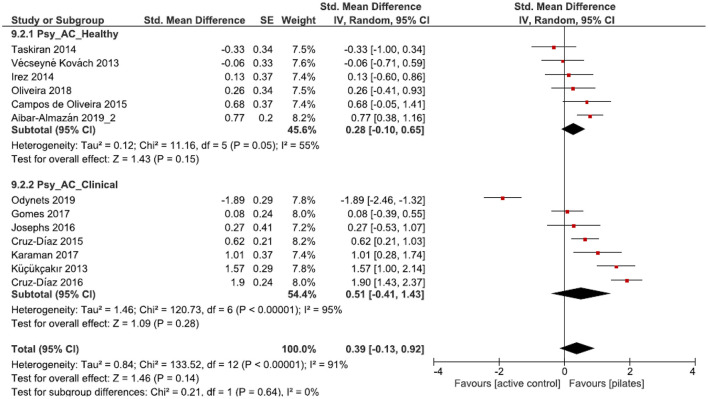
Forest plot of standardized mean effects of Pilates intervention on psychological health parameters compared to active control groups. Data are presented for healthy older adults and older adults with clinical conditions. SE, standard error; IV, inverse variance model; CI, confidence interval; Std., standardized.

Compared to ACs, small to moderate and significant effect sizes were found for the physiological health parameters [muscle strength, balance, endurance, flexibility, and physical functioning] in both groups [*p* = 0.04, SMD: 0.27 (95% CI: 0.02, 0.53), l^2^ = 55%; *p* = 0.01, SMD: 0.50 (95% CI: 0.10, 0.90), l^2^ = 68%; Figure 6], revealing more favorable outcomes for PIs than for ACs. No subgroup differences were observed [*p* = 0.35, SMD: 0.36 (95% CI: 0.14, 0.57), l^2^ = 60%; Figure 6]. Considering psychological health parameters (quality of life, depression, sleep quality, fear of falling, and pain), both subgroups were characterized by no significant effects [*p* = 0.15, SMD: 0.28 (95% CI: −0.10, 0.65), l^2^ = 55%; *p* = 0.28, SMD: 0.51 (95% CI: −0.41, 1.43) l^2^ = 95%; Figure 7]. Further, no subgroup differences were found [*p* = 0.64, SMD: 0.39 (95% CI: −0.13, 0.92), l^2^ = 91%; Figure 7].

## Discussion

Pilates is known to be a suitable fitness and rehabilitation approach for older adults (19, 103, 104). Whereas, the effects of Pilates on physiological parameters are well-documented (20), less is known about the impact on psychological health outcomes (44). Also, systematic reviews examining the overall (clinical) effectiveness of Pilates on physiological and psychological health are still lacking (20). Although Pilates is already commonly used as a rehabilitation approach (25, 103, 104), systematic reviews examining the health effects of PIs in this field are missing (21, 25). Hence, this meta-analysis and systematic review aimed at investigating the pooled effects of PIs on both physiological and psychological health parameters and to additionally compare any effects between older adults with and without clinical conditions. Moreover, it set out to make precise and uniform statements on the training modalities.

### Physiological Health Parameters

Compared to ICs, PIs led to moderately larger improvements on physiological health parameters in both groups (clinical and non-clinical). The influence of PIs on individuals' physical condition has already been studied in detail and confirm the presented effects (19, 105). Nevertheless, a higher amount of unspecific physical activity has been found to be effective and contribute to overall health in the older population (20, 103, 104, 106). Exercise therapy is, therefore, firmly established in the clinical field and subject to ongoing research (107). When considering the moderate improvements of PIs over ICs, the general effectiveness of physical exercising must be regarded.

Small effects were detected when comparing PIs in healthy older adults to ACs. Hence, PIs seem slightly better in improving physiological health than other exercise-based interventions. Notably, 17 out of 33 studies including healthy participants used an AC as a comparison, whereas former reviews stated the absence of studies with active control conditions (19, 103). Thus, the present review provides an additional evidence base for the need to investigate the effects of Pilates vs. other intervention approaches. Small effects were already reported in existing research, supporting the efficacy of PIs in physical abilities when compared to ACs (25).

The effects in patients with a clinical condition were moderate and high when compared to ACs. Thus, Pilates seems to be more effective in the clinical field when compared to other exercise therapies. These findings are in line with earlier investigations, in which Pilates led to greater improvements in patients with chronic low back pain than other exercise-based approaches (108). Significant improvements were obtained regarding physiological health parameters. Regardless of the control conditions, Pilates improved many physiological health measures of older adults. Unlike other reviews, ACs were not neglected in the present work. The moderately larger improvements in the clinical area of PIs over ACs imply a substantial higher usefulness of PIs in rehabilitation than in the fitness context. However, no significant subgroup differences were shown.

Nevertheless, these results should not be overstated. In five cases, AC training was also carried out by the Pilates group (e.g., physiotherapy, home-based exercises, conventional stroke therapy), so that Pilates can be considered a supplemental therapy approach (55, 61, 62, 74, 77). Seven studies used different exercise approaches that were only performed by the AC (e.g., traditional exercising, Yoga, physical activity program) (69, 73, 75, 83, 84, 97, 99). It is therefore proposed that the clinical effectiveness of Pilates, independent of its role as a supplementary therapy, should be further investigated and compared to other independent methods.

### Psychological Health Parameters

Maintaining the psychological health of older adults is of high importance (109). Besides the loss of physical abilities and structures, the aging process and supplemental (chronic) diseases have a significant influence on individuals' psychological health (13). Other mind-body interventions (MBI) (e.g., Tai Chi or Qi Gong) have already been examined with respect to their effects on psychological health factors in healthy older adults or the management of chronic diseases (110–112). Thus, Pilates was expected to positively influence psychological health parameters in older adults with and without clinical conditions. The included studies show moderate improvements over ICs in healthy older adults and large effects in older adults with a clinical condition. Besides the investigation of other MBIs, Pilates has already been shown to be suitable in improving quality of life, sleep quality, happiness, depression, and mood states in older adults (1, 19, 91, 113). In a younger population, Pilates had significant and positive effects on depressive symptoms, anxiety, feelings of energy, and fatigue when compared to ICs and regardless of individuals' health status (44).

The positive influence of social contacts, which arise during group training, should not be neglected (114). Especially in the case of inactive control groups, this influence should be balanced out by a socializing factor. This was, however, only mentioned in two studies (63, 64). Additionally, the statistical power of the clinical effects is limited by the small number of included studies. Future RCTs need to take this aspect into account and should provide a larger evidence base for further meta-analysis. For both conditions (non-clinical and clinical), no significant effects have been found when compared to an AC. Considering the relatively small number of included studies for these comparisons, the present results should not be overstated. This meta-analysis is nonetheless consistent with previous findings and reinforces the previous state of knowledge. Therefore, it becomes imperative to thoroughly investigate the psychological health parameters associated with PIs for both conditions (19, 42).

Previous findings indicate beneficial effects in pain, fatigue, and disability in participants with a clinical condition. In breast cancer, PIs led to higher improvements than home-based exercises (115) or other therapy approaches (33). Regarding the clinical conditions of diabetes type 2, Pilates exercises led to a significant reduction in glycated hemoglobin and oxidative stress (116). In patients with different clinical conditions, PIs decreased pain and disability symptoms in contrast to ICs and ACs (25). Similarly, Pilates reduced pain in patients with chronic low back pain more than usual care or short-term physical activity did (108). The included studies, which investigated pain symptoms, support these findings. All three studies showed that PIs reduced pain more than ACs (61, 62, 75). In the rehabilitative context, PIs seem to be promising, although the number of studies that investigated its effectiveness in older adults is limited. In general, an increased susceptibility to (chronic) diseases in old age and associated psychological health burdens (13) make examinations in the clinical and rehabilitative field essential (19, 25, 44). Furthermore, the included studies demonstrated a high degree of heterogeneity. This heterogeneity does not only relate to the different sample sizes, but also to the observed parameters, measurement instruments, and study qualities. Reducing the heterogeneity of the included studies would increase the comparability of PI studies (117) and, therefore, facilitate the interpretation of the obtained results.

Nevertheless, these results support recent studies. Pilates has been shown to be a safe and effective exercise intervention in adults over 50 years with various chronic conditions. In addition to physiological health improvements, a significant improvement in psychological factors was also demonstrated (9, 118). For future studies, it would nevertheless be useful to include a younger control group to provide information about whether the benefits of PIs are age dependent or not.

### Intervention

The included interventions had a average duration of 12–14 weeks for clinical and non clinical groups, respectively, mostly adopting two formal sessions per week of 60 min each. The consensus reached by most of the included studies is consistent with previous findings and can be described as follows: In terms of a positive change in physiological health parameters, two to three sessions per week for 1–6 months and 50–60 min of exercise per each session were considered sufficiently (20, 23, 103, 105). Greater effects were achieved when interventions lasted 24–36 sessions (20). In contrast, little is known about the intervention characteristics related to potential changes in psychological health parameters. Bullo et al. (19) regarded 12 weeks of intervention as sufficient to improve depressive symptoms. Positive effects in psychological health parameters of healthy older adults have already been obtained in this meta-analysis, but the small number of studies and the comparison to ICs must be considered. Hence, it is not possible to determine precisely the optimum intervention length enhancing psychological health. Based on previous research and the average length of the included interventions, an intervention length of minimum 12 weeks (9, 19) should be provided in the future.

The investigation of the training modalities and described intensities also demonstrated considerably large heterogeneity. Due to the different training approaches and exercise routines, no definite recommendations can be made. It is notable that most studies (irrespective of participants' clinical condition) focused on the implementation of mat-based Pilates. This might be due to the simple and cost-saving modality of mat-based Pilates, especially in the context of training in larger groups (2). A recent study confirms that mat and apparatus Pilates training did not significantly differ and led to similar physiological improvements in older women (119). By describing Pilates as a low- to moderate-intensity workout in its fundamental concepts (104), any future Pilates interventions should adhere to these intensity levels. Despite the different intervention types, Pilates can positively influence physiological and psychological health parameters in older adults with and without clinical conditions. For better comparability and testing of the effects, uniform exercise guidelines should be defined, which applies to psychological parameters as well as the rehabilitative context in general.

Pilates is described as a safe and suitable exercise method (2, 19, 113) for different populations (25). This statement is supported by the fact that none of the included studies reported any adverse events or accidents. Only three studies (77, 84, 100) have made clinical exercise adjustments, supporting the notion that Pilates can easily be applied and individualized for a heterogeneous and older target group.

### Strengths and Limitations

Due to the large numbers of included studies (*n* = 51), which tested a total of more than 2,000 participants, a structured, valid, and quantitative review of the current scientific evidence was presented. To ensure a high methodological standard, only randomized controlled trials were considered in our meta-analysis. A crucial advantage of this analysis lies in the comparison between the pooled effects of PIs on physiological and psychological health parameters in older adults. Another key aspect is the division into two target groups, allowing an analytical and critical comparison of the fitness- and clinical-related application of PIs. Focusing on a clinical population allows a closer and more realistic examination of the present situation, as around 50% of older adults suffer from two or more chronic diseases (37). This finding is in line with previous propositions stating that exercise therapies must be effective as well as applicable to a heterogenous population (19, 39). Also, the reference to a clinical population relies on elaborations of previous studies that stressed this lack of investigation as a limitation and an avenue for further investigations (19, 23, 25, 42, 44).

Despite the advantages of this meta-analytical work, some limitations need to be addressed. First, the search strategy and used inclusion criteria refer exclusively to English and German language publications or available text translations. A potential language bias can, therefore, not be excluded. Additionally, the search was limited to three databases, meaning that not all potential publications or unpublished trials were considered. Second, the broad range of included studies entails heterogeneity within the analysis, as demonstrated in parts of our results. Large differences were evident with regard to sample sizes, length and frequency of the intervention, measured health parameters, measurement techniques, and the use of active or inactive control groups. This heterogeneity as well as the lack of intervention descriptions (e.g., intensity, exercise description) prevents standardization or generalizability of training recommendations for Pilates. Accordingly, no significant differences in training modalities could be found between healthy participants and participants with a clinical condition. In terms of generalizability, best-practice recommendations in Pilates potentially engage in more precise results and facilitate individual adaptations (29). In addition, the heterogeneity in study designs and measured parameters prevented the separate consideration and comparison of specific individual physiological and psychological health parameters. Future studies should examine the respective individual factors in more detail to enhance the understanding of the various health outcomes of Pilates training. Third, within IC comparisons, the general effectiveness of physical activity (103, 104, 106) and the positive socializing factor of group training must be taken into account (114). In several cases, ICs served as control comparisons. Especially in the clinical area, where sufficient studies are still lacking, this factor can be stated as a limitation due to missing comparisons with other exercise therapies. Exercise therapies should be implemented as an AC in the future to evaluate the potential of clinical-based PIs and to gain further knowledge about the extent of PIs' effectiveness in relation to other exercise approaches.

## Conclusion

Since age-related declines in physical and psychological function is marked by several impairments, suitable training interventions engaging in overall fitness and resulting in physiological and psychological health are needed. Pilates is a promising and multimodal approach, but is lacking a systematic, overall investigation of its effects in different settings. Irrespective of older adults' health condition, PIs led to beneficial effects in physiological and psychological health parameters. Minor effects were demonstrated when PIs were compared with other exercise approaches. No differences on PIs' effectiveness were found depending on older adults' health condition (clinical or non-clinical).

Interpreting these results, it becomes evident that the current state of research inconclusive with respect to the effects of PIs on psychological health parameters, the investigation of PIs in a clinical setting, as well as the comparison of PIs to other exercise approaches. Nonetheless, Pilates seems to be a promising and safe therapy approach, especially for older adults with and without a clinical condition. The dominant large heterogeneity in study designs and training modalities impedes the formulation of valid best-practice recommendations for clinical- and non-clinical interventions. Therefore, future studies need to address the different training modalities carefully and differentiate between different intervention settings. In addition, assorted domains of research, such as studies examining the effects of PIs in older adults' psychological health or the comparison to a younger target group, should be explored further to yield a more thorough and comprehensive understanding of PIs' full potential in this context. Still, Pilates interventions already are an important low-cost, non-pharmaceutical, and effective method for an inherently heterogeneous target group.

## Data Availability Statement

The raw data supporting the conclusions of this article will be made available by the authors, without undue reservation.

## Author Contributions

LM: conceptualization, methodology, software, validation, formal analysis, investigation, resources, data curation, writing—original draft, and visualization. PW: writing—review and editing and supervision. LD: conceptualization, methodology, software, validation, formal analysis, investigation, resources, data curation, writing—review and editing, project administration, and supervision. All authors have read and agreed to the published version of the manuscript.

## Funding

We acknowledge the financial support of the German Research Foundation (DFG) and the Open Access Publication Fund of Bielefeld University for the article processing charge.

## Conflict of Interest

The authors declare that the research was conducted in the absence of any commercial or financial relationships that could be construed as a potential conflict of interest.

## Publisher's Note

All claims expressed in this article are solely those of the authors and do not necessarily represent those of their affiliated organizations, or those of the publisher, the editors and the reviewers. Any product that may be evaluated in this article, or claim that may be made by its manufacturer, is not guaranteed or endorsed by the publisher.
