# Supplementary material for: Effects of Pilates Training on Physiological and Psychological Health Parameters in Healthy Older Adults and in Older Adults With Clinical Conditions Over 55 Years: A Meta-Analytical Review
Source: Front Neurol. 2021 Oct 25;12:724218. doi: 10.3389/fneur.2021.724218 (PMC8574969; doi:10.3389/fneur.2021.724218)
Supplement: Supplementary file 1 [file Data_Sheet_1.ZIP › Suppl. tables/Supplementary Table1_Studycharacteristics.docx]

Supplementary Table 1 *Study characteristics and overview of the included studies (n = 51).*

| Reference | Study Design +  Condition  (clinical vs.  non-clinical) | Sample (mean age ± SD) | Groups  (n) | Pilates intervention  (exercises, intensity,  and used equipment) | Inactive Control Group | Active Control Group (exercises, intensity,  and used equipment) | Training | Outcome | PEDro |
| --- | --- | --- | --- | --- | --- | --- | --- | --- | --- |
| Aibar-Almazán et al.  (2019) | 2-armed RCT (non-clinical) | only women P = 70.0 ± 7.8 IC = 67.0 ± 10.1 | N = 107 P = 52 IC = 55 | Mat Pilates - strengthening and stretching exercises for main body segments (10 repetitions (reps) per exercise); used equipment: resistance bands, rings, balls; adjustment of intensity for every subject | normal activity-level |  | 12 weeks,  2x per week,  60 min/session total: 24 sessions adherence: 79% | balance confidence (Activities-specific Balance Confidence Scale -ABC: level of confidence in performing a specific task);  fear of falling (Falls-Efficacy Scale International FES-I to evaluate fear of falling); postural control (Romberg Test - standing position with eyes open and closed for 30 seconds (s); Center Of Pressure (COP) movements: mediolateral and anterioposterior displacement, mean displacement and velocity of COP movement) | 11 |
| Aibar-Almazán et al. (2019) (2) | 2-armed RCT  (non-clinical) | only women P = 70.0 ± 7.8  IC = 66.8 ± 10.1 | N = 107 P = 52 IC = 55 | Mat Pilates - strengthening and stretching standing exercises of progressive intensity (10 reps per exercise), then: exercises with bands, Magic Circles, and Fitballs on mat to improve strength, resistance, and flexibility | normal activity-level |  | 12 weeks,  2x per week,  60 min/session total: 24 sessions adherence: 75% | sleep quality (Pittsburgh Questionnaire - PSQI: 19 self-rated questions to evaluate subjective sleep quality over the period of 1 month);  fatigue (Fatigue Severity Scale - FSSS: self-report questionnaire to measure fatigue with 9 items);  anxiety and depression (Hospital Anxiety and Depression Scale - HADS: to evaluate anxiety and depression and consists of 14 items) | 10 |
| Alvarenga et al.  (2018) | 3-armed RCT (non-clinical) | only women P = 65.4 ± 3.3  (AC = 65.4 ± 4.5) IC = 73.3 ± 5.5 | N = 36 P = 11 (AC = 11) IC = 9 | Equipment-based Pilates - Cadillac, Chair, and Reformer; 9 exercises per session with 1-3 sets and 12 reps; individual progress and intensity through adjusting of the springs | no exercises/ training or | Inspiratory Muscle Training – two times a week: 30 inspiratory efforts in 2 sets; 50% effort (increase by 10% every two weeks) – this study arm got categorized as inactive group and was excluded by the authors | 10 weeks,  2x per week,  45 min/session total: 20 Pilates sessions, 20 Inspiratory Muscle Training sessions adherence: 85% | respiratory muscle strength (measured with analog manovacuometer: max. inspiratory pressure, max. expiratory pressure); pulmonary capacity (measured with powerbreath device: pulmonary pressure, power, flow, energy) -  aerobic capacity (Six-minute Walking Test 6MWT - walking on a 30 m runway as far as possible in 6 min);  upper-body muscle strength (Curl up Test - counting the amounts of abdominal curl ups in one minute) | 7 |
| Appell et al. (2012) | 2-armed, inter-group semi - experimental RCT  (non-clinical) | P = 69.6 ± 3.1  AC = 69.7 ± 2.9 | N = 39 P = 19 AC = 20 | Mat Pilates - exercises in supine or standing position for challenging trunk and pelvic muscles and for balance (10 reps per exercise) |  | Unspecific activity program - flexibility, strengthening, aerobic exercises with low intensity and short sport games | 10 weeks,  2x per week,  60 min/session total: 20 sessions adherence: 80% | balance battery (Balance Test - GGT: 14 items regarding static and dynamic balance; items are arranged in order of increasing difficulty) | 6 |
| Barker et al. (2016) | 2-armed pilot RCT (clinical - risk at fall) | P = 69.2 ±6.7  AC = 69.4 ± 5.8 | N = 42 P = 20 AC = 22 | Equipment-based Pilates - used equipment: Reformer, Trapeze, Wunda Chair, Chi Ball, bands and foam roller; intensity was individually tailored, but progressive; individual difficulty level: 7 out of 10 (10 = most difficult) + home exercises (see active control group) |  | only home exercise (focus on balance and strengthening and performed in a standing position) | 12 weeks,  2x per week,  60 min/session + every day 20 minutes Pilates sessions at home total: 24 official sessions adherence: 75% | dynamic balance  Step Test (15 s to place feet on a step and back; counting the steps);  Functional Reach Test (weight shifting in a standing position);  Lateral Reach Test (like functional reach test, but to the left and right side);  Timed up and Go Test (TUG - standing up from a chair, walk 3 meters (m), turn, walk back to chair and sit again);  Dynamic Gait Index (8 items to assess gait quality); Four Square Step Test (measures the speed of rapid stepping in a squared format);  static balance Modified clinical Test of Sensory Interaction on Balance (30 s standing with eyes closed/open on firm or compliant surface) lower-body muscle strength (30s Sit to Stand - standing up and sitting back again on a chair as fast as possible); lower-body flexibility  Knee to wall Test (ankle flexibility - standing in front of a wall and try to reach the wall with the knee, increase the distance between foot and wall);  Straight leg raise Test (hamstring flexibility - passive test by flexing the patients hip with a straight leg in a lying position) | 8 |
| Bertoli et al. (2018) | 2-armed, within study control (non-clinical) | only women ALL = 62.0 ± 3.0 (no further information) | N = 28 P = 14 IC = 14 | Mat Pilates - used equipment: small balls, Swiss Ball, Gym Sticks, resistance bands; increase in difficulty and repetitions (from 6 to 10 reps.); 3 sets per exercise | within group control (normal activitylevel) |  | 12 weeks,  3x per week,  60 min/session total: 36 sessions adherence: 75% | lower-body muscle strength (knee & hip extensor and flexor strength measured with a dynamometer - peak torque: knee extensors isometric contraction, dynamic concentric & eccentric contractions for flexors and extensors; hip - isometric contractions for flexor muscles, dynamic test with concentric & eccentric contractions for flexors and extensors) | 5 |
| Bird et al. (2012) | 2-armed RCT with crossover design (non-clinical) | ALL = 67.3 ± 6.5 | N = 27  P = 14  IC = 13 | Equipment-based Pilates - Reformer and mat exercises; focus on balance and lower-limb strength; individual increasement of intensity through load and repetitions; copy of mat exercises for home exercises | normal activity-level |  | 5 weeks, 2x per week (+ 1x per week at home), 60 min/sessiontotal: 10 official sessionsadherence: 80% | static balance (COP sway - standing for 30s with eyes open or closed on a firm surface or a medium-density foam cushion); dynamic balance (Four Square Step Test); physical functioning (TUG);lower-body muscle strength (knee extensor strength - weighted knee extension; ankle dorsiflexors strength - measured with a mobile footplate, patients had to raise the forefoot as forcefully as possible without rising with the heel from the ground) | 7 |
| Campos de Oliveira  et al. (2015) | 2-armed RCT  (non-clinical) | P = 63.6 ± 1.0  AC = 64.2 ± 0.8 | N = 32 P = 16 AC = 16 | Equipment-based Pilates - 20 strengthening and stretching exercises for the main body segments; equipment used: Combo Chair, Cadillac Trapez, Reformer, Ladder Barrel; all exercises: 1 set with 10 reps; progress to the individual ability (but no more than 10 reps) |  | Static Stretching - 20 exercises for neck and upper limbs, lateral chain stretches and lower limbs, 3 sets of one repetition for 30 s; sitting, lying or standing position | 12 weeks,  2x per week,  60 min/session total: 24 sessions adherence: not mentioned | lower-body muscle strength (strength of knee extensors + flexors - measured with a isokinetic dynamometer, maximum (max.) voluntary contraction of knee extensory followed by max. voluntary contraction of knee flexors); balance battery (Berg Balance Scale (BBS) - 14 tasks of daily living with 5 respond options to obtain the balance ability);  functional mobility (TUG);  health related Quality of Life (short-form health survey (SF-36) - 36 items referring to physical functioning, pain, health state, vitality etc. with a max. of 100 points and to measure quality of life) | 8 |
| Carrasco-Poyatos et al. (2019) | 3-arm quasi-experimental RCT  (non-clinical) | P = 67.5 ± 3.9  AC = 73.4 ± 4.8  IC = 65.9 ± 4.5 | Women N = 47 P = 16 AC = 19 IC = 12 | Mat Pilates - moderate to moderate-to-vigorous intensity; dynamic range of motion exercises, Pilates exercises, static range of motion and breathing exercises | normal activity-level | Muscular Strength Training - moderate to moderate-to-vigorous intensity; 3 parts: dynamic range of motion, muscular strength, static range of motion, and breathing exercises | 18 weeks,  2x per week,  60min/session total: 36 sessions adherence: 80% | functional autonomy (GDLAM - 5 tests of daily living such as 10m walking speed and standing up from a seated position; time is recorded for every test);  static balance (Two-leg Stance Test - eyes open, measuring 30 seconds the amplitude of displacement of COP in anterior-posterior plane) | 7 |
| Carrasco-Poyatos et al. (2019) (2) | 3-armed quasi-experimental RCT  (non-clinical) | only women P = 67.5 ± 3.9  AC = 73.4 ± 4.8 IC = 65.9 ± 4.5 | N = 60 P = 16 AC = 19 IC = 14 | Mat Pilates - 2-week familiarization, four 4-week mesocycle for increasing demands; moderate to moderate-to-vigorous intensity; focus on spine, hip, and girdle regions, exercising arm and legs & Pilates principles | normal activity-level | Resistance Training -familiarization, then increasing demands; moderate to moderate-to-vigorous intensity; focus on spine, hip, and girdle regions, exercising arms and legs | 18 weeks,  2x per week,  60min/session total: 36 sessions adherence: 80% | muscle strength (trunk and hip isometric and hip isokinetic strength - assessed with dynamometer);  static balance (one leg standing, single task condition - time in position, displacement velocity in media-lateral and anterior posterior, velocity moment);  dynamic balance (TUG) | 9 |
| Cruz-Díaz et al. (2015) | 2-armed RCT (clinical - chronic low back pain) | only women P = 72.8 ± 3.5  AC = 69.6 ± 2.2 | N = 97 P = 47 AC = 50 | Mat Pilates - plus physiotherapy; no more information were given |  | Physiotherapy - for all: 6 weeks, 2x per week; Transcutaneus Electrical nerve therapy (40 min), massage and stretching of low-back (20 min) | 6 weeks, 2x per week, 60 min/session total: 12 Pilates sessions (+ 12 therapy sessions) adherence: 75% | fear of falling (FES-I); functional mobility and balance (TUG);  pain intensity (NSR - a scale to measure pain intensity from 0 - 10) | 8 |
| Cruz-Diaz et al. (2016) | 2-armed RCT (clinical - chronic low back pain) | only women P = 69.6 ± 2.2  AC = 72.7 ± 3.5 | N = 101 P = 53 AC = 48 | Mat Pilates - plus physical therapy; strengthening, flexibility and joint mobility exercises; equipment used: Fitballs, Magic Rings, Thera Bands; breathing and motor control exercises; intensity increased through exercise variation |  | Physical Therapy - analgesic electrotherapy - (40 min); joint mobilization of the lumbar spine (10 min); 6 weeks, 2x per week | 6 weeks,  2x per week,  60 min/session total: 12 Pilates sessions (+ 12 physical therapy sessions) adherence: 85% | pain intensity (NSR);  functional impairment (Oswestry disability Index ODI - 10-items questionnaire to rate functional status according to Low Back Pain) | 7 |
| Curi et al. (2018) | 2-armed RCT (non-clinical) | only women P = 64.2 ± 0.1  IC = 63.7 ± 0.1 | N = 61 P = 31 IC = 30 | Mat Pilates - power house exercises in lying and standing positions, variation of strengthening exercises; intensity increased after two weeks - intermediate level exercises were added | no activities (monthly group meetings for communi-cation) |  | 16 weeks,  2x per week,  60 min/session total: 32 sessions adherence: not mentioned | sleep quality (Pittsburgh questionnaire - PSQI);  general health perception (General Health Questionnaire GHQ-12 - 12-item questionnaire, rated on a four-point scale to rate mental health) | 7 |
| Curi et al. (2018) (2) | 2-armed RCT  (non-clinical) | only women P = 64.2  IC = 63.7 (SD was not mentioned) | N = 61 P = 31 IC = 30 | Mat Pilates – power house exercises in lying and standing positions, variation of strengthening exercises; intensity increased after two weeks - intermediate level exercises were added | no activities (monthly group meetings for communi-cation) |  | 16 weeks,  2x per week,  60 min/session total: 32 sessions adherence: not mentioned | functional autonomy (Senior Fitness Test SFT - assessment of lower limb strength, upper limb strength, upper and lower body flexibility, dynamic balance, aerobic endurance);  life satisfaction (brazilian version of the Satisfaction with Life Scale; 5 items and rated on a 7-point Likert scale and to measure subjective well-being) | 6 |
| de Oliveira et al. (2019) | 2-arm RCT (clinical - chikungunya fever) | P = 54.4 ± 10.6 IC = 59.6 ± 9.4 | N = 42  P = 22  IC = 20 | Mat Pilates + standard clinical care - light-to-moderate intensity (from 6 to 12 reps); exercises involved coordination, strength, flexibility, and balance; used equipment: Swiss Ball, elastic bands; 18 to 22 exercises per session | standard clinical care |  | 12 weeks, 2x per week, 50 min/session  total: 24 sessions  adherence: not mentioned | pain intensity (measured with Visula Analogue Scale VAS, score from 0 to 10); flexibility (joint range of motion measured with goniometer - shoulder, wrist, knee, ankles, cervical + lumbar spine);functioning capacity (Health assessment questionnaire HAQ - to assess fine-motor movements of upper extremities, locomotor activities of lower extremities, and activities with both); quality of life (SF-12 - short-form of the SF-36) | 8 |
| Dlugosz-Bos et al. (2021) | 2-arm RCT (non-clinical) | only women  P = 67.7 ± 4.1  IC = 68.1 ± 3.3 | N = 50  P = 30  IC = 20 | Mat Pilates - exercises involving balancing, strengthening or stabilizing the body, breathing exercises, from standing to lying, additional balance and coordination exercises, 10 reps for each exercise | normal activity-level |  | 12 weeks,  2x per week,  45 min/session  total: 24 sessions  adherence: not mentioned | functional fitness (TUG);  static balance (One Leg Stance Test (OLS) - measured with force plate, left and right side single leg stance with eyes open for 30 seconds - COP total path length displacement; measuring of surface of the ellipse, length of displacements and mean velocity of the COP in one leg stance (each leg) for 10 seconds; Limits of Stability (LoS) – tests the ability to maintain COP outside the plain of support, maximum angle at which a participant was able to tilt away without loosing balance) | 7 |
| Donath et al. (2015) | 3-arm RCT  (non-clinical) | P = 70.8 ± 6.5 AC = 69.1 ± 5.8 IC = 69.9 ± 10.6 | N = 48 P = 17 AC = 16 IC = 15 | Mat Pilates - structure and exercise sequences remained constant; mobilization, breathing and axial extension in suspine, prone plank, side plank, quadruped, or sitting position; 6 - 12 reps per exercise (increasing intensity) | normal activity-level | Traditional Balance Training - in accordance with the Pilates training program in terms of duration, total amount of repetitions, number of sets, number exercises, progression and training intensity; exercises to train static and dynamic postural control | 8 weeks,  2x per week,  66 min/session total: 16 sessions adherence: 80% | static balance (OLS);  dynamic balance (Y-Balance Test: standing on the right/left foot reaching in the anterior direction followed by the trials standing on the left/right foot reaching in the anterior direction, repeated for the posteromedial and the posterolateral directions);  postural control (Perturbed Kneeling: measured on straingauge-based system - to examine reflectory dynamic control with perturbation - COP path length displacement);  upper-body muscle strength (ACSM Curl up Test: roll up into a crunch position and hold the position until exhaustion, time is reported; dynamic trunk strength endurance: performance of repetive crunches in a certain frequency until exhaustion, repetitions are counted) | 8 |
| Fourie et al. (2013) | 2-armed RCT (non-clinical) | only women P = 66.1 ± 4.8  IC = 65.3 ± 5.0 | N = 50 P = 25 IC = 25 | Mat Pilates - familiarization with the exercise program; sessions: breathing exercises, flow of movements from standing, to sitting, to lying down | normal activity-level |  | 8 weeks,  3x per week,  60 min/session total: 24 sessions adherence: not mentioned | flexibility (measured with gravity-based Leighton Flexometer in a lying position - shoulder flexion + extension, hip flexion, knee flexion) | 5 |
| Fretta et al. (2021) | 2-armed RCT (clinical – breast cancer) | only women  N = 55.3 ±10.9  P = 53.3 ± 8.6  IC = 57.5 ± 13.0 | N = 34 P = 18 IC = 16 | Mat Pilates – exercises were adapted to the individual ability (upper limb movements; 10 min warm-up, 40 min main part, 10 min cool-down) | educa-tional workshops (stret-ching, self-esteem, body image) |  | 16 weeks,  3x per week,  60 min/session  Total: 48 sessions  adherence: 68% | balance (Mini BestTest – 14 items testing balance disorder, from low to high points) | 7 |
| Gabizon et al. (2016) | 2-armed RCT (non-clinical) | P = 70.3 ± 3.8  IC = 72.1 ± 4.6 | N = 78 P = 34 IC = 44 | Mat Pilates - Level 1: exercises in a lying position, activation of core muscles; Level 2: core muscles and balance exercises on swiss balls for balance; Level 3: sitting exercises + strength exercises (with TheraBand), addresses balance of upper body and postural control | no exercises/ training |  | 12 weeks,  3X per week,  60 min/session total: 36 sessions adherence: n = 9: 86–97%;  n = 11: 34–85%;  n = 14: 3–33% | static balance (postural stability examination: quiet standing, feet together with eyes open or closed for 30 seconds - mediolateral and anterior posterior sway range, mean velocity and sway area);  balance battery (BBS) | 7 |
| Gomes et al. (2018) | 3-armed RCT (clinical - post-prostatec-tomy urinary inconti-nence) | only men P = 66.6 ± 5.7  AC = 65.8 ± 5.6 IC = 63.1 ± 7.2 | N = 104 P = 34 AC = 35 IC = 35 | Mat Pilates - 10 exercises with 10 reps each; exercises changed after 5 weeks for increasement of intensity, focus on pelvic region and trunk stabilization; 3 exercises as home workout | no exercises/ training | Pelvic Floor Muscle Training with Anal Electrical Stimulation (AES) - AES for 20 min and then followed by voluntary contractions of the pelvic floor muscles (PFM) in a lying, sitting, and standing position; daily PFM training at home | 10 weeks,  1x per week,  45 min/session + daily exercises at home total: 10 official sessions  adherence: not mentioned | pelvic floor muscle strength (measured with perineometry - max. strength, endurance, power);  quality of life (ICIQ-SF - to evaluate the frequency, severity, and impact on quality of life of urinary incontinence in men and women) | 9 |
| Hyun et al. (2014) | 2-armed RCT (non-clinical) | only women P = 70.0 ± 2.2  AC = 69.3 ± 2.6 | N = 40  P = 20 AC = 20 | Mat Pilates - breathing, strengthening, and stretching exercises |  | Unstable Support Surface Exercise - balance exercises on an aero-step, e.g. adapting to base of support, bipedal stance and moving the center of the body | 12 weeks,  3x per week,  40 min/session total: 36 sessions adherence: not mentioned | static balance (Romberg's Test: measured on Biorescue - COP sway length, sway average speed; eyes open, bipedal stance); dynamic balance (TUG) | 6 |
| Irez et al. (2011) | 2-armed RCT (non-clinical) | only women P = 72.8 ± 6.7 IC = 78.0 ± 5.7 | N = 60 P = 30 IC = 30 | Mat Pilates - additional Thera-Band elastic resistance exercises, Pilates ball exercises for beginners (Stott Pilates Comprehensive Mat work Manual); color of elastic bands for increasing intensity | normal activity-level |  | 12 weeks,  3x per week,  60 min/session total: 36 sessions adherence: 80% | dynamic balance (measured with dynamic stability measurement platform in a standing position for 30 sec. on an unstable surface);  lower-body muscle strength (measured with Muscle Manual Tester - hip flexion, hip abduction, hip adduction);  flexibility (Sit-and-Reach Test - reaching with the fingertips to the toes with one hand and in a sitting position) | 7 |
| Irez et al. (2014) | 3-armed RCT(non-clinical) | <65 (no further information) | N = 45  P = 15  AC = 15  IC = 15 | Mat Pilates - modified Pilates-based mat exercises, TheraBandexercises and Pilates Ball exercises for beginners with low- and moderate-intensity | no exercises/training | Walking - at low- to moderate intensity; no more information are given | 14 weeks, 3x per week, 60min/sessiontotal: 42 sessionsadherence: 80% | balance battery (BBS);balance confidence (ABC Scale); risk of falling (Downton Fall Risk Index - 11-item questionnaire to score risk of falling);muscle strength (hip flexion right/left and shoulder abduction right/left measured with a muscle tester) | 6 |
| Josephs et al. (2016) | 2-armed RCT  (clinical - impaired balance) | P = 75.6 ± 6.2  AC = 74.5 ± 6.9 | N = 24 P = 13 AC = 11 | Equipment-based Pilates - Reformer, Cadillac, Chair Apparatus; increased intensity through challenging exercises; 10 reps per exercise; 12-14 on Borg Perceived Exertion Scale |  | Traditional Exercising - equipment used: elastic resistance bands, ankle weights, foam balance pads, boxes, half foam rollers; repetitions of exercised were progressed individually, more than 20 reps - exercise difficulty was increased; 12-14 on Borg Scale | 12 weeks, 2x per week,  60 min/session (home-exercising on the other days 15-20 min) total: 24 official sessions adherence: 100% | physical functioning (TUG);  balance battery (FAB - includes challenging static and dynamic balance tasks and evaluates the balance performance); balance confidence (ABC Scale) | 8 |
| Karaman et al. (2017) | 2-armed RCT (clinical - total knee arthroplasty) | P = 67.6 ± 7  AC = 70.1 ± 6.9 | N = 34 P = 17 AC = 17 | Pilates Training + Standard Exercise Program - activation of transversus abdominus (TrA) and multifidus (Mf) muscles by using a Chattanooga stabilizer pressure biofeedback unit; strengthening hip and knee muscles, attain a maximum knee flexion angle, restore full knee extension |  | Standard Exercise Program - (3x per day, 5 min of patellar mobilization, isometric exercises every hour with 10 reps, isotonic strengthening exercises 3x per day with 5 reps), inflammation - reps were decreased; 2. week: standing while exercising, 3. week: starting with resistance exercises to strengthen the hip and knee muscles | 6 weeks, 2x per week (no more information);  all patients were given an earlier orthopedic rehabilitation program | balance battery (BBS);  quality of life (SF-36) | 6 |
| Karimi et al. (2021) | 3-armed RCT (clinical – knee osteo-arthritis) | only women  P = 60 ± 4.6  AC = 61.4 ± 4.8  IC = 60 ± 4 | N = 30  P = 10  AC = 10  IC = 10 | Mat Pilates – posture Training, stretching, balance, respiratory, strength and endurance exercises; every 3 sessions the load was increased (reps) | no exercises/ training | Suspension training with TRX - posture Training, stretching, balance, respiratory, strength and endurance exercises applied with TRX; same training dose as Pilates Group; used heart rate as marker | 8 weeks,  3x per week,  60 min/session  total: 24 sessions  adherence: not mentioned | dynamic balance (Y-Test - three reaching directions with the feet while maintaining balance)  lower-body-flexibility (measure the range of motion of the knee with goniometer) | 6 |
| Küçükçakır et al.  (2013) | 2-armed RCT (clinical - osteo-porosis) | only women P = 56.6 ± 5.5  AC = 56.3 ± 5.0 | N = 60 P = 30 AC = 30 | Mat Pilates - antalgic and stretching exercises, proprioceptive training, and respiratory training; used equipment: exercise bands and balls |  | Home Exercise - thoratic extension exercises in sitting position were shown by a physiotherapist, patients had to perform 3 sets with 20 reps for 1 year | 1 year, 2x per week, 60 min/session total: circa 104 sessions adherence: not mentioned | pain (visual analogue scale - VAS); endurance (6-Minute Walk Test);  lower-body muscle strength (Sit-to-stand-Test - rise from a chair as quick as possible for 1 minute, repetitions are recorded); quality of life (Qualeffo-41 - osteoporosis specific questionnaire to evaluate quality of life & SF-36) | 9 |
| Lim et al. (2016) | 2-armed RCT (clinical - post stroke) | P = 66.8 ± 5.7 IC = 61.1 ± 6.6 | N = 19 P = 10 IC = 9 | Mat Pilates - to improve core stability, breathing exercises in a sitting position: 1 set with 8 reps; spine mobility, upper limb, and lower limb strengthening exercises, additional gluteal activation | no exercises/ training |  | 8 weeks,  3x per week,  60 min/session total: 24 sessions adherence: not mentioned | static balance (measured with force plate under a treadmill, bipedal stance with open eyes for 30 seconds, COP sway in medio-lateral, anterior-posterior direction and velocity); dynamic balance (measured with force plate under a treadmill, walking on the treadmill at self-selected speed, COP sway in medio-lateral, anterior-posterior direction and velocity) | 7 |
| Lim et al. (2017) | 2-armed RCT (clinical - post stroke) | P = 63.2 ± 7.9 AC = 62.1 ± 6.7 | N = 20 P = 10 AC = 10 | Mat Pilates + Rehabilitation program - exercises were modified to be suitable for stroke patients, equipment used: balls, Magic Circles, and TheraBand; breathing exercises to improve core stability, spine mobility exercises, upper limb and lower limb strengthening exercises |  | only  Conventional Stroke Rehabilitation Program - joint mobility, strengthening, walking (no further information) - for 8 weeks, 5x per week,  30 min/session | 8 weeks, 3x per week, 60 min/session total: 24 sessions adherence: not mentioned | endurance (measured with treadmill and radio wire metabolic analyzer; treadmill exercise test: Harbor protocol - starts with the usual walking speed, for 10 minutes by increasing 0.09 m/s (0.2 mph) every 2 minutes until 80% of max. heart rate); Vo2 max + Vo2 max per kg and (ml/min));  physical functioning (TUG) | 8 |
| Liposcki et al. (2019) | 2-armed RCT (non-clinical) | only women P = 63.7 ± 3.3  IC = 65.2 ± 3.0 | N = 20 P = 9 IC = 11 | Equipment-based Pilates - focus on strength, flexibility, and balance of paravertebral, abdominal, and lower limb musculature; used equipment: Cadillac, Reformer, Chair, and mats; second phase: increased resistance and intensity, then maintenance of resistances | normal activity-level |  | 6 months,  2x per week,  30 min/session total: circa 62 sessions adherence: 90% | quality of life (SF-36) | 7 |
| Marinda et al. (2013) | 2-armed RCT(non-clinical) | only women  P = 66.1 ± 4.8  IC = 65.3 ± 5.0 | N = 50P = 25IC = 25 | Mat Pilates - increasing intensity during intervention period; first breathing, then flowing system from standing, to sitting, to lying down exercises, ended with rest position | no exercises/training |  | 8 weeks, 3x per week, 60 min/sessiontotal: 24 sessionsadherence: not mentioned | cardiometabolic parameters (resting heartrate - measured in a seated position after 5 min of rest; resting blood pressure - measured in a lying position after 5 min of rest) | 6 |
| Markovic et al. (2015) | 2-armed parallel-group RCT (non-clinical) | only women ALL = 70 ± 4.0 (no further information) | N = 30  P = 14 AC = 16 | Mat Pilates - core stability, quadruped exercises, kneeling and standing exercises, exercises for the upper and lower body with elastic bands; each exercise: 2-4 sets, 15-20 s contraction time or 15-20 reps; progress in terms of repetitions and load of exercises at the earliest opportunity |  | Huber Training - Training on a Huber device; mobility exercises, combined core and balance exercises, first two weeks: force level 50% of max. voluntary contraction (MVC), 65% MVC during the next 3 weeks, 75% of MVC during the last 3 weeks; increased intensity through additional core and balance perturbations | 8 weeks,  3x per week,  60 min/session total: 24 sessions adherence: 90% | static balance (measured with a force plate, semi-tandem stance and additional simple or dual tasks; 30 s trials - mean COP velocity, mean COP velocity in anterior-posterior and medio-lateral direction);  muscle strength (isometric strength of trunk extensors, flexors and lateral flexors measured with a dynamometer under static conditions; dynamic strength of upper body muscles measured with one repetition maximum on a bilateral chest press; leg muscle power: measured with the countermovement jump test, vertical jump height) | 7 |
| Melo et al. (2020) | 2-armed RCT (clinical - diabetes II) | only women P = 65.5 ± 5.5  IC = 67.5 ± 6.3 | N = 22 P = 11 IC = 11 | Mat Pilates - at moderate intensity, exercise modifications every 3 weeks; used equipment: swiss balls, elastic bands; dynamic and static stretching, general conditioning, relaxation; 1 set with 10 reps or 60 s hold | no further information |  | 12 weeks,  3x per week,  60 min/session total: 36 sessions adherence: 80% | functional autonomy (GDLAM - protocol) | 6 |
| Mesquita  et al. (2015) | 3-armed RCT (non-clinical) | only women P = 67.3 ± 4.9  AC = 68.5 ± 5.4 IC = 71.5 ± 6.2 | N = 58 P = 20 AC = 20 IC = 18 | Mat Pilates - range of motion and strength of upper limbs, trunk, and lower limbs; associated with contraction of transverse abdominal muscles and breathing; intensity was increased through swiss ball, TheraBand, and magic circle | normal activity-level | PNF (proprioceptive neuromuscular facilitation) - coordinative and proprioception activities; rhythmic initiation, sustentation and relaxation, reversal of antagonist; hold-relax stretching technique on lower and upper limbs; intensity was raised from 1 to 3 sets with 10 reps per exercise | 4 weeks,  3x per week, 50min/session total: 12 sessions adherence: 85% | functional mobility (TUG);  dynamic balance (Functional Reach Test);  balance battery (BBS);  static balance (bipedal stance, eyes open, for 30s - COP: total displacement of oscillation, anterior posterior and mid-lateral displacement movement, displacement area, average speed of mid-lateral and anteroposterior, average speed, total average speed) | 7 |
| Mollinedo-Cardalda et al. (2018) | 2-armed RCT  (clinical - Parkinson stage 1-3) | P = 62.8± 9.7  AC = 66.0 ± 13.1 | N = 26 P = 13 AC = 13 | Mat Pilates with TheraBand - 7 exercises with 3 sets of 8 reps; 7 on Modified Borg Rating of Perceived Exertion; increased intensity through more resistance |  | Physical Activity Program - combination of aerobic exercises e.g. strengthening, dynamic stretching, and coordination tasks; most of the exercises in a standing position | 12 weeks,  2x per week,  60 min/session total: 24 sessions adherence: 80% | lower-body muscle strength (30 s Chair Stand Test; Five Times Sit to Stand Test: rise and sit back on a chair for five times as fast as possible);  dynamic balance (TUG); | 6 |
| Odynets et al. (2019) | 3-armed RCT (breast cancer) | only women P = 59.4 ± 1.2  AC (1) = 58.8 ± 1.4 AC (2) = 59.1 ± 1.4 | N = 115 P = 40 AC (1) = 45 AC (2) = 30 | Mat Pilates - with resistance bands; first and second degree of lymphedema: exercises in standing, lying on the back, lying on the side, and sitting on the Fitball position; third degree of lymphedema: majority of exercises from lying initial positions without special equipment; intensity: 45 to 60% of heart rate reserve |  | Water Exercises (AC 1) - (not included in study data) Yoga (AC 2) - Hatha Yoga approach; asana techniques, breathing exercises, from static to dynamic asana complexes; exercise varied from low to moderate | 1 year,  3x per week, 60 min/session total: 144 sessions adherence: 90% | quality of life (Functional Assessment of Cancer Therapy Questionnaire - FACT-B: specific questionnaire for breast cancer patients to rate the quality of life) | 8 |
| Oksuz et al. (2017) | 2-armed RCT(clinical - osteo-porosis) | only women  P = 59.4 ± 7.5  IC = 61.0 ± 7.5 | N = 40P = 20IC = 20 | Clinical Pilates - increase in difficulty over the trial e.g.: progresses from closed-to open-chain kinetic exercises (no further information) | normal activity-level |  | 6 weeks, 3x per week, 60 min/sessiontotal: 18 sessionsadherence: not mentioned | fear (Tampa Kinesiophobia Scale - TKS to measure fear of avoidance in movement); pain (VAS; pain description (pain, pain level during study, total pain level) with 6-point Likert scale; Pain disability Index (PDI - to measure the influence of pain on daily life)); Oswestry Low Back Pain Disability scale (ODI) balance battery (BBS); functional balance (TUG); lower-body muscle strength (30 s Chair Sit and Stand Test); lower-body flexibility (Chair sit and reach Test); upper-body flexibility (Back scratch test); quality of life (QUALEFFO-41); anxiety and depression (Hospital Anxiety and Depression Scale - HADS);chronic functional impairment (Health Assessment Questionnaire); life satisfaction (Satisfaction with Life Scale) | 7 |
| Oliveira et al. (2016) | 2-armed RCT (non-clinical) | only women P = 63.6 ± 1.0 AC = 64.2 ± 1.0 | N = 32 P = 16 AC = 16 | Equipment-based Pilates - dynamic stretching and strengthening; with Chair, Cadillac Trapeze, Universal Reformer, Ladder Barrel; 10 reps per exercise, intensity was increased through position changing |  | Static Stretching - 20 static stretching exercises which cover the main body segments (lower and upper body, trunk); 3 sets per exercise and 30 s hold in lying, sitting or standing positions | 12 weeks,  2x per week,  60 min/session total: 24 sessions adherence: 80% | flexibility (measured with fleximeter - trunk flexion movement, trunk extension movement, hip flexion movement, plantar flexion movement of the ankle, dorsiflexion movement of the ankle) | 9 |
| Oliveira et al. (2017) | 2-armed RCT (non-clinical) | only women P = 65.1 ± 3.7 IC = 66.3 ± 3.4 | N = 30 P = 15 IC = 15 | Equipment-based Pilates - Reformer, Wall Unit, Cadillac Trapeze, Chair and Ladder Barrel; stretching, and strengthening of upper and lower body; 1 set with 10 reps per exercise; intensity was increased through position changing, sets and repetitions remained | normal activity-level |  | 12 weeks,  2x per week,  60 min/session total: 24 sessions adherence: 80% | upper-body muscle strength (elbow flexor and extensor muscle strength measured with isokinetic dynamometer - peak torque & total work);  functional performance of upper limbs (Put on and take off T-shirt Test - to verify the functionality of the upper limbs) | 8 |
| Oliveira et al. (2017) (2) | 2-armed RCT (non-clinical) | only women P = 63.6 ± 1.0  AC = 64.2 ± 0.8 | N = 32 P = 16 AC = 16 | Equipment-based Pilates - Chair, Cadillac Trapeze, Reformer, Ladder Barrel; one set, 20 stretching and strengthening exercises, 10 reps each to target major body segments; individual increasement of intensity (change of position or higher resistance); moderate level of effort |  | Static Stretching - 20 static stretching exercises for cervical region and upper limbs, trunk and lower limbs, 3 series, maintaining for 30 s in exercise; in a lying or standing position | 12 weeks,  2x per week,  60 min/session  total: 24 sessions adherence: 80% | lower-body muscle strength (knee extensor and flexor muscle strength in both limbs was measured with an isokinetic dynamometer; max. voluntary contraction, peak torque, total work) | 9 |
| Oliveira et al. (2018) | 3-armed RCT (non-clinical) | only women P = 55.5 ± 6.8 AC = 56.3 ± 6.4 IC = 54.1 ± 5.2 | N = 51 P = 17 AC = 17 IC = 17 | Equipment-based Pilates - Cadillac, Reformer, Ladder Barrel, Wall Unit, Chair, Spine Corrector, Small Barrel; 21 strengthening and stretching exercises for the main body segments; 1 set with 10 reps per exercise; individual increasement of intensity through change of position or higher resistance; between 5 and 6 on Borg Scale | normal activity-level | Vibration - 5 min of whole-body vibration on a vibratory platform (20 Hz) | 6 months,  3x per week,  60 min/session total: 78 sessions (AC: 5 min/session) adherence: 90% | lower-body muscle strength (knee extensors and flexors muscle strength measured with an isokinetic dynamometer - max. force of voluntary contraction, peak isokinetic torque); quality of life (SF-36) | 8 |
| Patti et al. (2021) | 2-armed RCT (non-clinical) | P = 63.9 ± 4.4  AC = 63.3 ± 4.4 | N = 41  P = 18  AC = 23 | Mat Pilates – exercises from basic to intermediate level; 30 s or 5-20 reps per exercise; breathing, stretching, strengthening, and mobilisation exercises |  | General physical activity program - nonstandardized and equipment-free exercise program, included a sequence of stretching exercises and  aerobic exercise (160 min of moderate-intensity aerobic  activity each week for 13 weeks) | 13 weeks,  3x per week,  50 min/session  total: 39 sessions  adherence: not mentioned | postural control (Romberg Test – open eyes);  balance battery (Berg Balance Scale (BBS);  grip strength (dominant hand using an electronic dynamometer) | 8 |
| Roh et al. (2016) | 2-armed RCT (clinical - poststroke) | ALL = 66.1 ± 4.4 (no further information) | N = 20 P = 10 IC = 10 | Mat Pilates - breathing, stretching, and strengthening exercises | no exercises/ training |  | 8 weeks,  3x per weeks,  60 min/session total: 24 sessions adherence: not mentioned | gait parameters (measured with a 3-D motion system with 8 infrared cameras on a treadmill - stride length, stride time, stride velocity, step length, hip range of motion (ROM), knee ROM, ankle ROM) | 3 |
| Siqueira Rodrigues et al. (2010) | 2-armed RCT (non-clinical) | only women  ALL = 66 ± 4  (no further information) | N = 52  P = 27  IC = 25 | Equipment-based Pilates - with Bobath Ball, Cadillac, Wall Unit, Combo Chair, Reformer; initialglobal stretching, general conditioning; increased intensity through higher resistance or change of position; 10 reps per exercise | no exercises/  training |  | 8 weeks,  2x per week,  60 min/session  total: 16 sessions  adherence: not mentioned | personal autonomy (GDLAM protocol); balance battery (Tinetti Test, test performances which are specific to measure balance in older adults);quality of life (WHOQOL-OLD - version for the elderly of the World Health Organisation's Quality of Life, 24 questions with 6 domains) | 7 |
| Sofianidis et al. (2017) | 3-armed RCT (non-clinical) | P = 70.8 ± 5.4  AC = 70.6 ± 5.8 IC = 70.4 ± 6.0 | N = 36 P = 12 AC = 12 IC = 12 | Mat Pilates - adapted to the age of the participants and the purpose of the intervention; used equipment: power balls, different diameters, ribbons-tires and weights of 1 kg (no further information) | no exercises/ training | Latin Dance - learning of Latin dances (e.g. Tango, Rumba, Bachata) appropriate for their age with or without partner (no further information) | 12 weeks,  2x per week,  60 min/session total: 24 sessions  adherence: not mentioned | static balance (2-leg Tandem stance with open and closed eyes and 1-leg stance with open eyes to measure COP displacement on a 3D force plate during static balance conditions);  dynamic balance (measuring COP displacement on a 3D force plate during rhythmic sway in anterior posterior direction - voluntarily shifting) | 4 |
| Taskiran et al. (2014) | 3-armed RCT (non-clinical) | P = 76.2 ± 7.5 AC = 77.2 ± 6.4  IC = 80.0 ± 6.2 | N = 58 P = 18 AC = 18 IC = 22 | Mat Pilates - used equipment: exercise bands, free weights; breathing, stretching and mobility exercises, Pilates and strengthening exercises | normal activity-level | Yoga - degree of difficulty and duration of the asanas was increased according to the capacity of the subjects; each week 7 poses (maintaining for 4 s at the beginning and then increased to 12 s at the end of the intervention) | 8 weeks,  3x per week,  50 min/session total: 24 sessions adherence: 83% | quality of life (Nottingham health profile - NHP: 38 items which address pain, physical mobility, emotional reactions, energy, social isolation, and sleep dimensions);  grip strength (dominant hand using a vigorometer);  balance battery (BBS);  physical functioning (Senior Fitness Test - SFT: test battery to measure physical functioning in different domains (e.g. strength, flexibility, endurance)) | 6 |
| Teixeira de Carvalho  et al. (2017) | 3-armed controlled RCT (non-clinical) | ALL = 69.2 ± 6.1 (no further information) | N = 60 P = 20 AC = 20 IC = 20 | Mat Pilates - progressive: resistance, difficulty, and the number of repetitions increased gradually; muscle stretching of the upper limbs, trunk, and lower limbs, then exercises involving range of motion and strength of upper limbs, trunk, and lower limbs, breathing exercises; used equipment: Swiss ball, TheraBand, and Magic Circle | no exercises/ training | PNFG - progressive: resistance, difficulty, and the number of repetitions increasing gradually; stretching (hold-relax technique for upper and lower limbs); exercises for the upper limbs, in a bilaterally symmetrical pattern, and for the lower limbs in a asymmetric bilateral pattern; additional scapular and pelvic gridle exercises | 4 weeks,  3x per week,  50 min/session total: 12 sessions adherence: 84% | lower-body muscle strength (max. isometric force for knee extensors and flexors, measured with a load cell and a surface EMG System) | 7 |
| Tozim et al. (2020) | 3-armed controlled RCT (clinical – low back pain) | only women  P = 66.7 ± 3.6  AC = 68.5 ± 4.9  IC = 68 ± 4.7 | N = 46  P = 14  AC = 13  IC = 14 | Mat Pilates – used equipment: fitness ball, stretch band; progressive: through equipment, change of reps, less rest, and intermediate levels of exercises; stretching and strengthening exercises | edu-cational workshops (age changes, quality of life and physical activity) | General Exercise Group – same training load as Pilates Group; warm up (walking), main part (resistance and stretching exercises), cool-down (balance exercises); progressive through change of exercises) | 8 weeks,  2x per week,  60 min/session  total: 16 sessions  adherence: > 75% | lower-body flexibility (Chair sit and reach Test; Popliteal angle test (PA) – knee extension until sensation of muscle stretching in both legs);  upper-body muscle strength (trunk extension and with submaximal force, four seconds of max. isometric strength) | 8 |
| Vécseyné Kovách  et al. (2013) | 3-armed RCT (non-clinical) | P = 66.6 ± 5.5  AC = 67.9 ± 6.9 IC = 64.6 ± 6.2 | N = 54 P = 22 AC = 17 IC = 15 | Mat Pilates - some exercises were excluded from the Pilates training (e.g. exercises resulting in high chest pressure, burden the lumbar-sacral junction, and trunk rotational exercises) | no exercises/ training | Aqua fitness - shallow water training was applied, where the feet touch the bottom of the pool and water does not exceed shoulder level (no further information) | 6 months; 3x per week, 60 min/session total: 72 sessions adherence: 80% | physical functioning (Fullerton Functional Fitness Test to measure the ability to perform everyday activities);  quality of life (WHOQOL-OLD) | 5 |
| Vieira et al. (2017) | 2-armed RCT (non-clinical) | only women P = 66.0 ± 1.3  IC = 63.3 ± 0.9 | N = 40 P = 21 IC = 19 | Mat Pilates - used equipment: rubber bands, swiss and exercise balls; to work the full range of motion and to incorporate strength, balance and coordination in different body positions | normal activity-level |  | 12 weeks,  2x per week,  60 min/session total: 24 sessions adherence: 70% | static balance (One-leg Stance Test - eyes open);  physical functioning (TUG);  lower-body muscle strength (5-times-sit-to-stand test);  endurance (6-Minute-Walk Test) | 7 |
| AC, active control group; Hz, Hertz; IC inactive control group; min, minutes; P, Pilates intervention; RCT, randomized controlled trial | | | | | | | | | |
