# Supplementary material for: Effects of Pilates Training on Physiological and Psychological Health Parameters in Healthy Older Adults and in Older Adults With Clinical Conditions Over 55 Years: A Meta-Analytical Review
Source: Front Neurol. 2021 Oct 25;12:724218. doi: 10.3389/fneur.2021.724218 (PMC8574969; doi:10.3389/fneur.2021.724218)
Supplement: Supplementary file 1 [file Data_Sheet_1.ZIP › Suppl. tables/Supplementary Table2_PEDroScores.docx]

Supplementary Table 2 *PEDro criteria and sum of scores of the included studies.*

| **Author** | **year** | **eligability specified** | **subjects randomly allocated** | **concealed allocation** | **similar baseline values** | **blinding of subjects** | **blinding of therapist** | **blinding of assesor** | **dropout <15%** | **received treatment as allocated** | **statistcial between-group comparison** | **point measures and variability provided** | **sum (2 to 11)** |
| --- | --- | --- | --- | --- | --- | --- | --- | --- | --- | --- | --- | --- | --- |
|  |  | **1.** | **2.** | **3.** | **4.** | **5.** | **6.** | **7.** | **8.** | **9.** | **10.** | **11.** |  |
| **Aibar-Almazán et al.** | 2019 | √ | √ | √ | √ | - | - | - | √ | √ | √ | √ | **8** |
| **Aibar-Almazán et al._2** | 2019 | √ | √ | √ | √ | - | - | - | √ | √ | √ | √ | **8** |
| **Appell et al.** | 2012 | √ | √ | - | √ | - | - | - | √ | √ | √ | √ | **7** |
| **Barker et al.** | 2015 | √ | √ | √ | √ | - | - | - | √ | √ | √ | √ | **8** |
| **Bertoli et al.** | 2017 | √ | √ | - | - | - | - | - | - | √ | √ | √ | **5** |
| **Bird et al.** | 2012 | √ | √ | - | √ | - | - | √ | - | √ | √ | √ | **7** |
| **Campos de Oliveira et al.** | 2015 | √ | √ | √ | √ | - | - | - | √ | √ | √ | √ | **8** |
| **Carrasco-Poyatos et al.** | 2018 | √ | √ | √ | - | - | - | √ | - | √ | √ | √ | **7** |
| **Carrasco-Poyatos et al.** | 2019 | √ | √ | √ | √ | - | - | √ | - | √ | √ | √ | **8** |
| **Cruz-Díaz et al.** | 2015 | √ | √ | - | √ | - | - | √ | √ | √ | √ | √ | **8** |
| **Cruz-Díaz et al.** | 2016 | - | √ | √ | √ | - | - | - | √ | √ | √ | √ | **7** |
| **Curi et al.** | 2018 | - | √ | √ | √ | - | - | - | √ | √ | √ | √ | **7** |
| **Curi et al._2** | 2018 | - | √ | √ | - | - | - | - | √ | √ | √ | √ | **6** |
| **de Alvarenga et al.** | 2018 | √ | √ | √ | - | - | - | - | √ | √ | √ | √ | **7** |
| **de Andrade Mesquita et al.** | 2015 | √ | √ | √ | - | - | - | - | √ | √ | √ | √ | **7** |
| **de Oliveira et al.** | 2016 | √ | √ | √ | √ | - | - | - | √ | √ | √ | √ | **8** |
| **de Oliveira et al._2** | 2016 | √ | √ | √ | √ | - | - | √ | √ | √ | √ | √ | **9** |
| **de Oliveira et al.** | 2019 | √ | √ | √ | √ | - | - | √ | - | √ | √ | √ | **8** |
| **Dlugosz-Bos et al.** | 2021 | √ | √ | - | √ | - | - | - | √ | √ | √ | √ | **7** |
| **Donath et al.** | 2015 | √ | √ | √ | √ | √ | - | - | - | √ | √ | √ | **8** |
| **Fourie et al.** | 2013 | - | √ | - | √ | - | - | - | √ | √ | - | √ | **5** |
| **Fretta et al.** | 2021 | √ | √ | - | √ | - | - | √ | - | √ | √ | √ | **7** |
| **Gabizon et al.** | 2016 | √ | √ | √ | √ | - | - | √ | √ | √ | √ | √ | **9** |
| **Gomes et al.** | 2017 | √ | √ | √ | √ | - | - | √ | √ | √ | √ | √ | **9** |
| **Hyun et al.** | 2014 | √ | √ | - | - | - | - | - | - | √ | √ | √ | **5** |
| **Irez et al.** | 2011 | √ | √ | - | √ | - | - | √ | - | √ | √ | √ | **7** |
| **Irez et al.** | 2014 | √ | √ | - | √ | - | - | - | √ | √ | - | √ | **6** |
| **Josephs et al.** | 2016 | √ | √ | √ | √ | - | - | √ | - | √ | √ | √ | **8** |
| **Karaman et al.** | 2017 | √ | √ | - | √ | - | - | - | - | √ | √ | √ | **6** |
| **Karimi et al.** | 2021 | √ | √ | - | √ | - | - | - | - | √ | √ | √ | **6** |
| **Küçükçakır et al.** | 2013 | √ | √ | √ | √ | - | - | √ | √ | √ | √ | √ | **9** |
| **Lim et al. 2016** | 2016 | √ | √ | - | √ | - | - | - | √ | √ | √ | √ | **7** |
| **Lim et al. 2017** | 2017 | √ | √ | √ | √ | - | - | - | √ | √ | √ | √ | **8** |
| **Liposcki et al.** | 2019 | √ | √ | √ | √ | - | - | - | - | √ | - | √ | **6** |
| **Marinda et al.** | 2013 | - | √ | √ | √ | - | - | - | √ | √ | - | √ | **6** |
| **Markovic et al.** | 2015 | √ | √ | - | - | - | - | √ | √ | √ | √ | √ | **7** |
| **Melo et al.** | 2018 | - | √ | - | √ | - | - | - | √ | √ | √ | √ | **6** |
| **Mollinedo-Cardalda et al.** | 2017 | - | √ | √ | √ | - | - | - | - | √ | √ | √ | **6** |
| **Odynets et al.** | 2019 | - | √ | √ | √ | - | - | √ | √ | √ | √ | √ | **8** |
| **Oksuz et al.** | 2017 | √ | √ | - | √ | - | - | - | √ | √ | √ | √ | **7** |
| **Oliveira et al.** | 2017 | √ | √ | √ | √ | - | - | √ | √ | √ | √ | √ | **9** |
| **Oliveira et al.** | 2018 | √ | √ | √ | √ | - | - | - | √ | √ | √ | √ | **8** |
| **Patti et al.** | 2021 | √ | √ | √ | √ | - | - | √ | - | √ | √ | √ | **8** |
| **Roh et al.** | 2016 | - | √ | - | - | - | - | - | - | √ | - | √ | **3** |
| **Siqueira Rodrigues et al.** | 2010 | √ | √ | √ | - | - | - | - | √ | √ | √ | √ | **7** |
| **Sofianidis et al.** | 2017 | - | √ | - | - | - | - | - | - | √ | √ | √ | **4** |
| **Taskiran et al.** | 2014 | - | √ | - | √ | - | - | - | √ | √ | √ | √ | **6** |
| **Teixeira de Carvalho et al.** | 2017 | √ | √ | - | √ | - | - | - | √ | √ | √ | √ | **7** |
| **Tozim et al.** | 2020 | √ | √ | √ | √ | - | - | - | √ | √ | √ | √ | **8** |
| **Vécseyné Kovách et al.** | 2013 | - | √ | - | - | - | - | - | √ | √ | √ | √ | **5** |
| **Vieira et al.** | 2016 | √ | √ | √ | √ | - | - | - | - | √ | √ | √ | **7** |
